# Complete analytical solutions for double cantilever beam specimens with bi-linear quasi-brittle and brittle interfaces

**DOI:** 10.1007/s10704-018-0324-5

**Published:** 2018-11-14

**Authors:** Leo Škec, Giulio Alfano, Gordan Jelenić

**Affiliations:** 1 Department of Mechanical and Aerospace Engineering, Brunel University London, Kingston Lane, Uxbridge, UB8 3PH UK; 2 Faculty of Civil Engineering, University of Rijeka, Radmile Matejčić 3, Rijeka, 51000 Croatia

**Keywords:** DCB test, Mode-I delamination, Analytical solution, Timoshenko beam theory, Cohesive-zone model, Linear-elastic fracture mechanics

## Abstract

In this work we develop a complete analytical solution for a double cantilever beam (DCB) where the arms are modelled as Timoshenko beams, and a bi-linear cohesive-zone model (CZM) is embedded at the interface. The solution is given for two types of DCB; one with prescribed rotations (with steady-state crack propagation) and one with prescribed displacement (where the crack propagation is not steady state). Because the CZM is bi-linear, the analytical solutions are given separately in three phases, namely (i) linear-elastic behaviour before crack propagation, (ii) damage growth before crack propagation and (iii) crack propagation. These solutions are then used to derive the solutions for the case when the interface is linear-elastic with brittle failure (i.e. no damage growth before crack propagation) and the case with infinitely stiff interface with brittle failure (corresponding to linear-elastic fracture mechanics (LEFM) solutions). If the DCB arms are shear-deformable, our solution correctly captures the fact that they will rotate at the crack tip and in front of it even if the interface is infinitely stiff. Expressions defining the distribution of contact tractions at the interface, as well as shear forces, bending moments and cross-sectional rotations of the arms, at and in front of the crack tip, are derived for a linear-elastic interface with brittle failure and in the LEFM limit. For a DCB with prescribed displacement in the LEFM limit we also derive a closed-form expression for the critical energy release rate, $G_c$. This formula, compared to the so-called ‘standard beam theory’ formula based on the assumptions that the DCB arms are clamped at the crack tip (and also used in standards for determining fracture toughness in mode-I delamination), has an additional term which takes into account the rotation at the crack tip. Additionally, we provide all the mentioned analytical solutions for the case when the shear stiffness of the arms is infinitely high, which corresponds to Euler–Bernoulli beam theory. In the numerical examples we compare results for Euler–Beronulli and Timoshenko beam theory and analyse the influence of the CZM parameters.

## Introduction

Since introduced in 1960s by Dugdale (1960) and Barenblatt (1959), the use of cohesive-zone models (CZM) has become one of the most popular ways of describing fracture processes within the research community (Hillerborg et al. 1976; Alfano and Crisfield 2001; Park and Paulino 2011). Nowadays CZMs are widely implemented within the framework of finite-element analysis (FEA) and interface elements, based on CZMs, can be found in element libraries of many commercial softwares for FEA used to solve delamination/debonding problems in 2D (Alfano and Crisfield 2001) and 3D (Park and Paulino 2011). However, because the accuracy of such computations is always dependent on the size of the FE mesh, they can be computationally demanding and suffer from convergence problems.

One of the ways to reduce the computational cost of the analysis was already proposed by the first and third author and consists of using beam finite elements instead of plane solids to model the bulk material of the specimens in 2D analysis of delamination. The results were presented for single-mode (I and II) and mixed-mode delamination problems in geometrically linear (Škec et al. 2015) and non-linear analysis (Škec and Jelenić 2017). Compared to models which use plane solid FEs, the computational efficiency of the beam model was improved (the reduction of total number of degrees of freedom can go up to 40 %) without any significant loss in the accuracy. However, the beam model still suffered from convergence problems and spurious oscillations around the exact solution for cases of brittle interfaces when the mesh is not sufficiently refined. For this reason, it is always very useful when an analytical or semi-analytical solution can be found for cases of engineering interest.

In this paper we focus on mode-I delamination and the double cantilever beam (DCB) test, which is the standard test for determining fracture toughness in mode I. We use quasi-static and geometrically linear analysis where the arms of the DCB are modelled as Timoshenko beams and at the interface, a bi-linear CZM is used. Since the traction–separation law at the interface is composed of two linear parts and a final part with zero tractions, the solution of the problem can be obtained analytically, separately for each part of the interface whose state falls within one of the linear parts of the traction separation law. Because all the quantities of the problem will be expressed exactly with no need for discretisation, the computational cost is negligible and the obtained solutions have no spurious oscillations, which typically occur when delamination problems are solved using FE analysis (Alfano and Crisfield 2001; Blackman et al. 2003b; Škec et al. 2015). However, the idea of using analytical solutions for a DCB is not new and many researchers have contributed to the field in the last 50 years.

The simplest way to analytically model a DCB would be to assume that the arms of the specimen act as if they were cantilever beams clamped at the crack tip. Under this assumption, Benbow and Roesler (1957) used Euler–Bernoulli beam theory to establish the Griffith’s energy balance for a flat-strip specimen where the crack gradually propagates down the middle by holding the specimen in a state of lengthwise compression. During 1960s and early 1970s, Ripling et al. (1971) introduced the DCB and tapered double cantilever beam (TDCB) tests and specimens. They also provided analytical formulae based on Irwin’s energy approach (Irwin 1956) and Timoshenko beam theory (assuming that the arms are clamped at the crack tip) to compute the fracture toughness of the adhesive, which in 1974 became part of the American standard for determining fracture toughness of adhesive joints in mode I. The current version of that standard, ASTM D3433-99 (reapproved in 2012) (ASTM D3433-99 2012), still exclusively uses the same formulae proposed in Ripling et al. (1971). The formula for the DCB used in ASTM D3433-99 (2012) is also used in BS ISO 25217:2009 (2009), where it is called ‘simple beam theory’ (SBT). We will adopt this terminology and use the term ‘simple beam theory’ (SBT) for formulations based on simple beam theories (Euler–Bernoulli or Timoshenko) and the assumption that the DCB arms act as if they were clamped at the crack tip.

However, Ripling et al. (1971) noticed that the SBT formula gives smaller deflections than those obtained from the experiments and they attributed it to not taking into account the rotations of the arms which take place at the crack tip. They also suggested that the results could be simply corrected by increasing the measured crack length by a fixed amount. Although they did not propose a method to obtain this crack length correction, this concept was further developed by other researchers (Blackman et al. 2003a) and became the basis for a data reduction scheme in BS ISO 25217:2009 (2009) called ‘corrected beam theory’ (CBT) based on Euler–Bernoulli beam theory. de Moura et al. (2008) developed the so-called ‘compliance-based beam method’ (CBBM) where the corrected-crack-length concept was used with Timoshenko beam theory.

Kanninen (1973) presented an analytical model for a DCB where the upper arm was modelled as a Euler–Bernoulli beam on elastic foundation (Winkler model), allowing for relative displacements and rotations at the crack tip and ahead of it. The very next year (Kanninen 1974) extended his formulation to account for the shear deformability of the arms and rotational stiffness of the interface, which was accomplished by using Timoshenko beam theory and Pasternak elastic foundation. However, Gehlen et al. (1979) (with Kanninen as the third author) showed that the rotational stiffness of the foundation springs is not relevant in a symmetrical DCB configuration. Kanninen’s model (Kanninen 1974) was later extended by Williams (1989) to account for orthotropic material behaviour. Shahani and Forqani (2004), Shahani and Amini Fasakhodi (2010) developed solutions for a DCB model of finite length consisting of a Timoshenko beam lying on an elastic Winkler foundation for the conditions of fixed force and fixed displacement. Most of the mentioned papers (Kanninen 1973, 1974; Gehlen et al. 1979; Shahani and Forqani 2004; Shahani and Amini Fasakhodi 2010) also investigate the dynamic analysis of unstable crack propagation and arrest in a DCB test. We will refer to the beam-on-elastic-foundation DCB models as ‘enhanced beam theory’ (EBT) models.

In EBT models the interface acts elastically linear up to a certain point where brittle failure occurs. However, fracture processes can usually introduce a certain level of quasi-brittle behaviour, which cannot be captured using EBT models. It is, however, well know that the quasi-brittle behaviour of the interface in a DCB test has an important influence on the structural response before the crack starts to propagate, whereas during the crack propagation the influence of the interface ductility is negligible. One way of introducing a quasi-brittle behaviour of the interface in the model is to use CZMs which account for progressive softening/damage after a certain critical value of the traction at the interface has been reached.

Stigh (1988) developed an analytical solution for a DCB where the arms are modelled as Euler–Bernoulli beams and a bi-linear CZM is embedded at the interface. This solution was revisited and extended to account for a finite length of the specimen and a trapezoidal CZM by Dimitri et al. (2017). de Morais (2013) proposed a solution for a DCB with prescribed rotations (loaded with moments) where the arms are modelled as Timoshenko beams and a bi-linear CZM is embedded at the interface. Unlike Williams (1989) who gave the complete solution for the linear-elastic phase of the interface behaviour, de Morais (2013) took into account only real roots of the characteristic equation of the differential equation of the problem. We discuss this issue in detail in Sect. 2.2. We will refer to the models with quasi-brittle crack as ‘cohesive crack models’ (CCM) (Dimitri et al. 2017).

To the best of authors’ knowledge, a complete analytical solution for a DCB with arms modelled as Timoshenko beams and the interface modelled using a bi-linear CZM, which covers any of the two cases of prescribed rotations and displacement, is not available in the literature. Therefore, one aim of this paper is to fill this gap and to show a clear relationship between SBT, EBT and CCM solutions for a DCB for both Euler–Bernoulli and Timoshenko beam theory.

Furthermore, reducing the general CCM solution down to the SBT solution shows that even in the limit case of LEFM, rotations at the crack tip and in front of it still occur when Timoshenko beam theory is used. Thus, the assumption made in SBT that the arms act as if they were clamped at the crack tip is not applicable even for an infinitely stiff perfectly brittle interface, which is assumed in LEFM. This is somehow an expected result since even an infinitely stiff interface cannot prevent the bulk material of the arms to deform (rotate) around the interface. However, the fact that we can capture this behaviour using Timoshenko beam theory, to the best of authors’ knowledge, has not been addressed in the literature so far. More generally, a comprehensive investigation of the analytical solutions for the LEFM limit is lacking in the literature. Thus, we call this novel approach ‘enhanced simple beam theory’ (ESBT). However, we will show that when the shear deformability of the arms is excluded from the model, which corresponds to Euler–Bernoulli beam theory, ESBT is equivalent to SBT. A second aim of this paper is to determine the interface stresses and the stress resultant profiles in the LEFM cases. We will also show that a novel LEFM-based formula for the determination of the critical energy release rate, $G_c$, can be derived. This formula takes into account the rotation at the crack tip, unlike those currently available in the standards (ASTM D3433-99 2012; BS ISO 25217:2009 2009).

The outline of the paper is as follows. In Sect. 2, we define the problem and derive the general solutions of differential equations of the problem. In Sects. 3 and 4, we determine the integration constants for the cases of DCB with prescribed rotations and DCB with prescribed displacement, respectively. The solutions are given in a unified and compact form, thus avoiding cumbersome expressions which can be often encountered in such analytical solutions.

The presented general CCM solutions (based on Timoshenko beam theory and a bi-linear CZM at the interface) are then used to derive solutions for different particular cases. EBT solutions presented in Sect. 5 are obtained from CCM solutions by making the interface brittle (removing the softening branch in the traction–separation law of the interface), whereas ESBT (LEFM-based) solutions presented in Sect. 6 are obtained from EBT solutions by letting the interface stiffness go to infinity. The solutions for Euler–Bernoulli beam theory (including CCM, EBT and SBT solutions) given in “Appendix B” are obtained from the Timoshenko beam theory solutions by letting the shear stiffness become infinite.

Results obtained by means of the presented analytical solutions are studied for a number of cases, including sensitivity analyses on the interface strength. In particular, results for a linear interface with brittle behaviour and in the LEFM limit are presented and discussed in Sect. 7.3.

## Problem description

**Fig. 1:**
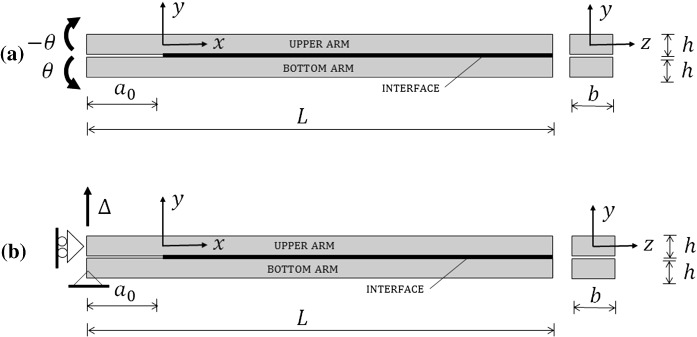
DCB with **a** prescribed rotations and **b** prescribed displacement

Consider a double cantilever beam (DCB) of length $L$ composed of two identical arms with depth $h$, width $b$ and the initial crack length ${a}_0$, as shown in Fig. 1. Each arm is modelled as a Timoshenko beam with a linear-elastic constitutive law, where material properties are defined by Young’s modulus, $E$, and shear modulus, $\mu$. In a general case $E$ and $\mu$ can have independent values, while for an isotropic material $\mu =0.5E/(1+\nu )$, where $\nu$ is Poisson’s ratio. Mode-I problem is considered, so that loads are applied symmetrically with respect to the mid-plane of the interface between the two arms. Therefore, stresses and strains in a DCB are symmetrical with respect to the mid-plane of the interface, which means that, for the sake of simplicity, only one arm can be considered in the analysis. In the present paper we will consider only the upper arm and assume that the *x*-axis is the centroidal axis of the arm (reference axis), while *y* and *z* axes are the principal axes of the arm’s cross section (see Fig. 1).

In our approach, Timoshenko beam theory is used to model the DCB arms, which implies that we assume that displacements and rotations of the arms are relatively small compared to the specimen’s dimensions. The edges of the beams (where the interface is attached) in the general case can move both in *x* and *y* directions. However, for our problem, which is symmetric with respect to the mid-plane of the interface, there is no relative displacement at the interface responsible for mode-II delamination. This is because the upper and the bottom arm of a DCB at the same co-ordinate *x* have the same, but opposite cross-sectional rotation. Since Timoshenko beam theory is a geometrically linear theory, all points in a single cross-section of the arm experience the same displacement in the *y*-direction, i.e. $v(x,y)=v(x)$. Therefore, for a DCB with symmetrical arm deformations, opening (mode-I) relative displacement at the interface, $\delta (x)$, corresponds to the sum of transverse displacements of both arms. This can be written as

1 $$\begin{aligned} \delta (x)=2\ v(x), \end{aligned}$$

where $v$ is the displacement of the upper arm. Note that in Fig. 1 the origin of the co-ordinate system is positioned at the crack tip, which means that at the left-hand end of the specimen $x=-{a}_0$.

The two types of tests we investigate in this paper are the DCB with prescribed rotations, $\theta$, and the DCB with prescribed displacement, $\Delta$, as shown in Fig. 1. In the first case the crack propagates with a constant cohesive zone length (i.e. crack propagation is steady-state) (Suo et al. 1992; Škec et al. 2018), the cohesive zone being where softening/damage of the interface takes place. In the second case the crack propagation is not steady-state, but it tends to being so in the limit of infinitely long cracks, i.e. as $a\rightarrow \infty$, where $a$ denotes the crack length. Complete solutions for both cases are given in Sects. 3 and 4, respectively.

**Fig. 2:**
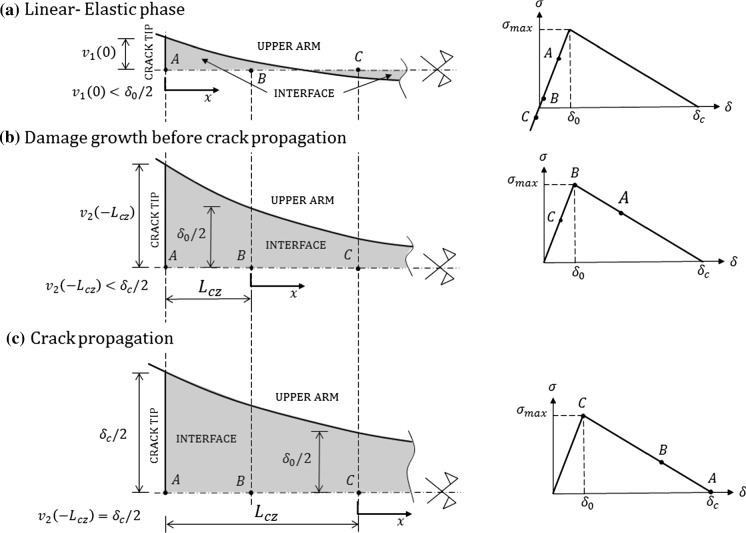
Three phases of the problem solution with the deformed shape of the interface (shaded in grey) given on the left-hand side, and the position of characteristic points (A, B and C) in the $\sigma -\delta$ diagram given on the right-hand side. Point A represents the initial crack tip ($a={a}_0$) in **a** and **b**, and the crack tip at any position where $a>{a}_0$ in **c**

For the interface we use a bi-linear CZM consisting of a linear-elastic branch and a linear softening branch, followed by zero tractions for relative displacements greater than the critical value ${\delta }_c$, as shown on the right-hand side in Fig. 2. Here we emphasise that CCM solutions presented in this paper are not general solutions valid for any shape of the traction–separation law of the CZM, but are only valid when the interface behaviour can be assumed as bi-linear with progressive damage. Thus, for a bi-linear CZM law the solution will be obtained for three different phases in the crack propagation process, which is explained in detail in the following subsection.

### Three-phase solution

In order to solve the problem of a DCB with an interface with embedded bi-linear CZM, the usual approach is to develop solutions for three different phases (Stigh 1988; de Morais 2013; Dimitri et al. 2017) which are namely: (i) linear-elastic behaviour, (ii) damage growth before crack propagation and (iii) crack propagation. The solution for each of these phases is derived in detail in the following sections according to Fig. 2, where only the upper half of the beam is shown. The origin of the co-ordinate system is in the left-most undamaged point for every phase, and it is moving to the right-hand side as the damage at the interface increases and the crack propagates. Transversal displacements of the upper arm are denoted by $v_1(x)$ for $x>0$ and by $v_2(x)$ otherwise for reasons which will be explained in Sect. 2.2.

#### Linear-elastic behaviour

In this phase the entire DCB acts as a linear-elastic body, which means that besides the DCB arms (with a linear-elastic constitutive law), the interface behaviour is linear-elastic, too. For all points at the interface undergoing separation (including points A and B in Fig. 2a), $\delta (x)<{\delta }_0$ and $\sigma (x)<\sigma _{max}$, where ${\delta }_0$ and $\sigma _{max}$ are linear-elastic limit values of the separation $\delta$ and the traction $\sigma$ on the interface, respectively. This also means that, as long as $\delta (x)<{\delta }_0$ and $\sigma (x)<\sigma _{max}$, no energy dissipation (damage) at the interface occurs and the initial (undeformed and undamaged) configuration can be recovered if the load is removed. Once at the crack tip $\delta (0)={\delta }_0$, we enter a new phase where damage at the interface starts to develop. This phase is explained in the following section.

It is well known that a zone of compressive stresses ($\sigma (x)<0$, resulting in $\delta (x)<0$) ahead of the crack tip exists in a DCB (Alfano and Crisfield 2001; de Morais 2015; Dimitri et al. 2017). However, our CZM assumes that in compression no softening (or damage) occurs, and the behaviour is still linear elastic. In Fig. 2a we can see that point C experiences negative (compressive) contact tractions and negative relative displacements at the interface. For a zero-thickness adhesive layer this results in a non-physical overlapping of the arms, which in our model is allowed and does not create any additional stresses. But because the interface thickness in our model does not influence the results (although it is well know that in reality a different thickness of the same adhesive results in a different structural behaviour) and we can assume that the interface is sufficiently thick to prevent the arms coming in direct contact.

#### Damage growth before crack propagation

In this phase, the interface can be divided in two zones. In the first zone, just ahead of the crack tip, damage is developing (although the crack is still not propagating) and $\delta (x)\in [{\delta }_0,{\delta }_c)$, where $x\in (-L_{cz},0]$. In the second zone interface behaviour is again linear-elastic, i.e. $\delta (x)<{\delta }_0$, where $x>0$. Note that according to Fig. 2b, $x=-L_{cz}$ corresponds to the initial crack tip and $x=0$ corresponds to the point where the first and the second zone meet. In this phase, the damage builds up with loading and $L_{cz}$ increases from 0 (corresponding to $\delta (-L_{cz})=\delta (0)={\delta }_0$) to a certain limit value (corresponding to $\delta (-L_{cz})={\delta }_c$) at which the crack begins to propagate. Note that, according to Fig. 2b, the $\sigma -\delta$ relationship in the first zone is also linear, but with softening (damage).

#### Crack propagation

This phase is similar to the previous phase (Sect. 2.1.2) with the difference that here the relative displacement at the current crack tip, $\delta (-L_{cz})={\delta }_c$, remains unchanged during the whole phase. For a DCB with prescribed rotations the cohesive zone length remains constant for any position of the crack (steady-state crack propagation), while for a DCB with prescribed displacement the cohesive zone length will change (non-steady-state crack propagation). Thus, when the crack propagation is steady state the deformed shape of the interface shown in Fig. 2c, and the contact traction distribution over the interface, $\sigma (x)$, simply translate to the right-hand side. This is why point A in Fig. 2c is the point at the interface where the crack tip is currently located. When the crack propagation is not steady-state, we still have $\delta (-L_{cz})={\delta }_c$ and $\delta (0)={\delta }_0$ for any position of the crack tip, but the deformed shape and the contact traction distribution at the interface (including $L_{cz}$) change as the crack propagates. The part of the interface which has been completely damaged ($\sigma (x)=0$) is excluded from the domain of the solution for $v_2(x)$ and becomes a part of the DCB arm.

### Solutions of differential equations of the problem

The differential equation of the Timoshenko beam reads

2 $$\begin{aligned} v^{\mathrm {IV}}(x)-\frac{1}{EI}q(x)+\frac{1}{{\mu }{A}_s}q^{\prime \prime }(x)=0, \end{aligned}$$

where $EI$ is the bending stiffness (with second moment of area $I=b\ h^3/12$), ${\mu }{A}_s$ is the shear stiffness (with corrected shear area $A_s=b\ h\ k_s$, where $k_s$ is the shear correction coefficient) and $q(x)$ is distributed transverse load along the beam axis, assumed to be positive when pointing upwards. Furthermore, in accordance with the co-ordinate system from Fig. 1, we have

3 $$\begin{aligned} q(x)&= {{\mathcal {T}}}^{\prime }(x), \end{aligned}$$

4 $$\begin{aligned} \varphi (x)&= v^{\prime }(x)+\gamma (x)=v^{\prime }(x)+\frac{{{\mathcal {T}}}(x)}{{\mu }{A}_s}, \end{aligned}$$

where ${{\mathcal {T}}}(x)$, $\gamma (x)$ and $\varphi (x)$ are the shear force, shear strains and cross-sectional rotation, respectively. We assume that the self-weight of the DCB arms is negligible compared to the magnitude of the external forces or moments acting on the DCB. Therefore, only contact tractions will act on the arms as a distributed load, and thus

5 $$\begin{aligned} q(x)=-b\ \sigma (x), \end{aligned}$$

where the negative sign appears because positive (tensile) contact tractions at the upper arm are pointed downwards. The part of the DCB arms separated by the initial crack, or where the interface has been completely damaged, is excluded from the domain of the solution $v(x)$. However, the moment or the force applied at the point of the prescribed rotation or displacement, respectively, (which is outside of the mentioned domain) are taken into account via the boundary conditions at the crack tip. This will be explained in detail in the following sections.

We define $\sigma (x)$ according to the traction–separation law of the CZM. Thus, in our case we will define $\sigma (x)$ separately for the linear-elastic ($\delta (x)\le {\delta }_0$) and for the linear softening part (${\delta }_0<\delta (x)\le {\delta }_c$) as

6 $$\begin{aligned} \sigma (x)=\left\{ \begin{array}{ll} \sigma _{max}\dfrac{\delta (x)}{{\delta }_0}, &{} \text {if }\delta (x)\le {\delta }_0,\\ \sigma _{max}\dfrac{{\delta }_c-\delta (x)}{{\delta }_c-{\delta }_0}, &{} \text {if }{\delta }_0<\delta (x)\le {\delta }_c. \end{array} \right. \end{aligned}$$

Substituting (6) in (5) and then in (2) and taking into account (1) we obtain two differential equations

7 $$\begin{aligned}&v^{\mathrm {IV}}(x)-2\ \omega \ \lambda ^2\ v^{\prime \prime }(x)\nonumber \\&\quad +\lambda ^4\ v(x)=0, \quad \text {if } v(x)\le \frac{{\delta }_0}{2}, \end{aligned}$$

8 $$\begin{aligned}&v^{\mathrm {IV}}(x)+2\ \psi \kappa ^2\ v^{\prime \prime }(x)-\kappa ^4 v(x)\nonumber \\&\quad +\kappa ^4\frac{{\delta }_c}{2}=0, \text {if } \frac{{\delta }_0}{2}<v(x)\le \frac{{\delta }_c}{2}, \end{aligned}$$

where

9 $$\begin{aligned}&\lambda =\root 4 \of {\frac{2\ b\ \sigma _{max}}{EI\ {\delta }_0}},&\omega =\frac{EI}{{\mu }{A}_s}\frac{\lambda ^2}{2}, \end{aligned}$$

10 $$\begin{aligned}&\kappa =\root 4 \of {\frac{2\ b\ \sigma _{max}}{EI\ ({\delta }_c-{\delta }_0)}},&\psi =\frac{EI}{{\mu }{A}_s}\frac{\kappa ^2}{2}. \end{aligned}$$

Equation (7) is used in all phases on the undamaged part of the interface ($x\ge 0$), while Eq. (8) is used only in phases 2 and 3 on the damaged part of the interface ($x\in [-L_{cz},0)$). We will denote the solutions of Eqs. (7) and (8) by $v_1(x)$ and $v_2(x)$, respectively, and derive them in the following sections.

#### Solution on the undamaged part of the interface

Assuming the solution of Eq. (7) in a form $v_1(x)=e^{r\ x}$, where *r* is a constant, results in a characteristic equation with four roots, namely

11 $$\begin{aligned} r_1=\lambda \ \zeta _1, \quad r_2=-\lambda \ \zeta _1, \quad r_3=\lambda \ \zeta _2, \quad r_4=-\lambda \ \zeta _2, \nonumber \\ \end{aligned}$$

where

12 $$\begin{aligned} \zeta _1=\sqrt{\omega +\sqrt{\omega ^2-1}},\quad \zeta _2=\sqrt{\omega -\sqrt{\omega ^2-1}}. \end{aligned}$$

Since $\omega \ge 0$, we will have all real roots for $\omega >1$, all complex roots for $\omega <1$ and multiple real roots for $\omega =1$. For each of these cases, we can now give:

1. The solution of Eq. (7) for $\omega >1$:
  13 $$\begin{aligned} v_1(x)= & {} e^{-\lambda \zeta _1 x}C_1+e^{-\lambda \zeta _2 x}C_2+e^{\lambda \zeta _1 x}{\overline{C}}_1\nonumber \\&+\,e^{\lambda \zeta _2 x}{\overline{C}}_2, \quad x\ge 0, \end{aligned}$$
  where $C_1$, $C_2$, ${\overline{C}}_1$ and ${\overline{C}}_2$ are integration constants.
2. The solution of Eq. (7) for $\omega <1$:
  14 $$\begin{aligned}&v_1(x) = e^{-\lambda \zeta _3 x}\left[ \sin (\lambda \ \zeta _4\ x)C_3+\cos (\lambda \ \zeta _4\ x)C_4\right] \nonumber \\&\quad +\,e^{\lambda \zeta _3 x}\left[ \sin (\lambda \ \zeta _4\ x){\overline{C}}_3+\cos (\lambda \ \zeta _4\ x){\overline{C}}_4\right] , \quad x\ge 0, \end{aligned}$$
  where
  15 $$\begin{aligned} \zeta _3= \sqrt{\frac{1+\omega }{2}}, \quad \zeta _4=\sqrt{\frac{1-\omega }{2}}, \end{aligned}$$
  and $C_3$, $C_4$, ${\overline{C}}_3$ and ${\overline{C}}_4$ are integration constants. Note that because of
  16 $$\begin{aligned} \zeta _1\ \zeta _2=1, \quad \text {and} \quad (\zeta _1+\zeta _2)^2=2(\omega +1), \end{aligned}$$
  we have
  17 $$\begin{aligned} \zeta _3=\frac{\zeta _1+\zeta _2}{2}, \quad \zeta _4=i\frac{\zeta _2-\zeta _1}{2}. \end{aligned}$$
3. The solution of Eq. (7) for $\omega =1$:
  18 $$\begin{aligned} v_1(x)= & {} e^{-\lambda x}(C_5+C_6\ x)+e^{\lambda x}({\overline{C}}_5\nonumber \\&\quad +\,{\overline{C}}_6\ x), \quad x\ge 0, \end{aligned}$$
  where $C_5$, $C_6$, ${\overline{C}}_5$ and ${\overline{C}}_6$ are integration constants.

In our approach we will assume that during crack propagation the crack tip is always sufficiently distant from the right-hand end of the DCB, in a way that any boundary conditions at the right-hand end (free or clamped) do not influence the results. This is equivalent to assuming a semi-infinite DCB. Therefore the domain of the undamaged part of the interface will have boundary conditions at $x=0$ and $x=\infty$, where we can write:

19 $$\begin{aligned} v_1(\infty )=0,\quad \varphi _1(\infty )=0, \end{aligned}$$

where $\varphi _1(x)$ is the cross-sectional rotation on the undamaged part of the interface. According to Timoshenko beam theory $\varphi _1(x)=v_1^{\prime }(x)+{{\mathcal {T}}}_1(x)/{\mu }{A}_s$, where ${{\mathcal {T}}}_1(x)$ is the cross-sectional shear force on the undamaged part of the interface. Because ${{\mathcal {T}}}_1(\infty )=0$, applying boundary conditions (19) to solutions (13), (14) and (18) gives ${\overline{C}}_i=0$, where $i=1,\ldots ,6$. Thus, the solution of Eq. (7) for a semi-infinite DCB can be written in a general form as

20 $$\begin{aligned} v_1(x)=\left\{ \begin{array}{ll} e^{-\lambda \zeta _1 x}C_1+e^{-\lambda \zeta _2 x}C_2, &{} \text {for }\omega >1,\\ e^{-\lambda \zeta _3 x}\left[ \sin (\lambda \ \zeta _4\ x)C_3+\cos (\lambda \ \zeta _4\ x)C_4\right] , &{} \text {for }\omega <1,\\ e^{-\lambda x}(C_5+x\ C_6), &{} \text {for } \omega =1, \end{array} \right. \nonumber \\ \end{aligned}$$

where $$x\ge 0$$.

##### Remark 2.1

For the sake of simplicity and because of the extreme unlikelihood that the value $\omega =1$ occurs in a real case, the solutions in the following sections are given only for the cases when $\omega >1$ and $\omega <1$. However, the results for $\omega =1$ are given separately in “Appendix A” for completeness. In the numerical examples presented in Sect. 7 we will show that, unlike stated in de Morais (2015), all solutions from Eq. (20) are possible for realistic values of geometrical and material properties of a DCB. $\square$

#### Solution on the damaged part of the interface

Assuming the solution of Eq. (8) in a form $v_2(x)=e^{tx}$, where *t* is a constant, results in a characteristic equation with four roots, namely

21 $$\begin{aligned} t_1=\kappa \ \xi _1, \quad t_2=-\kappa \ \xi _1, \quad t_3=i\ \kappa \ \xi _2, \quad t_4=-i\ \kappa \ \xi _2, \end{aligned}$$

where

22 $$\begin{aligned} \xi _1=\sqrt{-\psi +\sqrt{\psi ^2+1}}, \quad \xi _2=\sqrt{\psi +\sqrt{\psi ^2+1}}. \nonumber \\ \end{aligned}$$

Since $\xi _1$ and $\xi _2$ are real for any value of $\psi$, roots $t_1$ and $t_2$ are always real, whereas $t_3$ and $t_4$ are always imaginary. Thus, the solution of Eq. (8) can be written as

23 $$\begin{aligned} v_2(x)= & {} \sin (\kappa \ \xi _2\ x)D_1+\cos (\kappa \ \xi _2\ x)D_2\nonumber \\&+\sinh (\kappa \ \xi _1\ x)D_3+\cosh (\kappa \ \xi _1\ x)D_4+\frac{{\delta }_c}{2}, \nonumber \\ \end{aligned}$$

for $x\in [-L_{cz},0]$, where $D_i$, $i=1,\ldots ,4$ are integration constants.

In the following sections, the problem is solved and the integration constants are determined for each phase for a DCB with either prescribed rotations or prescribed displacement.

## DCB with prescribed rotations

Consider a DCB with prescribed rotations as illustrated in Fig. 1a. At the left-hand end of each arm an equal, but opposite rotation $\theta$ is prescribed, causing the opening of the DCB along the interface due to equal, but opposite concentrated moments, $M$ acting at the point of the prescribed rotation. This implies that the disconnected parts of the DCB arms are in pure bending and the shear force at the crack tip is zero during all phases. Each of the three solution phases is derived in detail in the following sections.

### Linear-elastic phase

Using (4), (5) and (6), we can express the cross-sectional bending moment, ${{\mathcal {M}}}_1(x)$ and shear force, ${{\mathcal {T}}}_1(x)$, in the upper DCB arm as:

24 $$\begin{aligned} {{\mathcal {M}}}_1(x)&= EI\ \varphi _1^{\prime }(x)=EI\left[ v_1^{\prime \prime }(x)-\frac{b\ \sigma (x)}{{\mu }{A}_s}\right] \nonumber \\&=EI\left[ v_1^{\prime \prime }(x)-2\ \omega \ \lambda ^2\ v_1(x)\right] , \end{aligned}$$

25 $$\begin{aligned} {{\mathcal {T}}}_1(x)&= {{\mathcal {M}}}_1^{\prime }(x)=EI\left[ v_1^{\prime \prime \prime }(x)-2\ \omega \ \lambda ^2v_1^{\prime }(x)\right] , \end{aligned}$$

where index 1 refers to the solution of Eq. (7), i.e. linear elastic behaviour. Using solutions (20), Eqs. (24) and (25) can be written as

26 $$\begin{aligned} {{\mathcal {M}}}_1(x)&= \left\{ \begin{array}{ll} -EI\ \lambda ^2\left( e^{-\lambda \zeta _1 x}C_1\ \zeta _2^2+e^{-\lambda \zeta _2 x}C_2\ \zeta _1^2\right) , &{} \text {for }\omega >1,\\ EI\ \lambda ^2\ e^{-\lambda \zeta _3 x}\left[ \sin (\lambda \ \zeta _4\ x)C_3^M+\cos (\lambda \ \zeta _4\ x)C_4^M\right] , &{} \text {for }\omega <1, \end{array} \right. \end{aligned}$$

27 $$\begin{aligned} {{\mathcal {T}}}_1(x)&= \left\{ \begin{array}{ll} EI\ \lambda ^3\left( e^{-\lambda \zeta _1 x}C_1\ \zeta _2+e^{-\lambda \zeta _2 x}C_2\ \zeta _1\right) , &{} \text {for }\omega >1,\\ EI\ \lambda ^3\ e^{-\lambda \zeta _3 x}\left[ \sin (\lambda \ \zeta _4\ x)C_3^T+\cos (\lambda \ \zeta _4\ x)C_4^T\right] , &{} \text {for }\omega <1, \end{array} \right. \end{aligned}$$

where

28 $$\begin{aligned} C_3^M&= C_3(\zeta _4^2-\zeta _3^2)+2\ C_4\ \zeta _3\ \zeta _4, \nonumber \\ C_4^M&= -2\ C_3\ \zeta _3\ \zeta _4+C_3(\zeta _4^2-\zeta _3^2), \end{aligned}$$

29 $$\begin{aligned} C_3^T&= C_3\ \zeta _3-C_4\ \zeta _4, \nonumber \\ C_4^T&= C_3\ \zeta _4+C_4\ \zeta _3. \end{aligned}$$

Please recall that the solutions for the case when $\omega =1$ are given in “Appendix A”. Using expressions (26) and (27), from the following boundary conditions at the crack tip:

30 $$\begin{aligned} {{\mathcal {M}}}_1(0)=M, \quad {{\mathcal {T}}}_1(0)=0, \end{aligned}$$

we obtain

31 $$\begin{aligned} C_1=\frac{M}{EI\ \lambda ^2(1-\zeta _2^2)}, \quad C_2=\frac{M}{EI\ \lambda ^2(1-\zeta _1^2)}, \end{aligned}$$

for $$\omega > 1$$, and

32 $$\begin{aligned} C_3=-\frac{M\ \zeta _3}{EI\ \lambda ^2\ \zeta _4}, \quad C_4=\frac{M}{EI\ \lambda ^2}, \end{aligned}$$

for $$\omega < 1$$.

The transition from the linear-elastic phase to the phase of damage growth before crack propagation, is defined by the condition

33 $$\begin{aligned} v_1(0)=\frac{{\delta }_0}{2}, \end{aligned}$$

for both $\omega >1$ and $\omega <1$ one has

34 $$\begin{aligned} v_1(0)=C_1+C_2=C_4=\frac{M}{EI\ \lambda ^2}. \end{aligned}$$

Hence, the moment $M_L$, leading to the transition from the first phase (linear-elastic behaviour) to the second phase (damage growth before crack propagation) reads

35 $$\begin{aligned} M_L=\frac{EI\ {\delta }_0\ \lambda ^2}{2}, \end{aligned}$$

for any value of $\omega$ (including $\omega =1$, as reported in “Appendix A”).

The relative displacement of the DCB arms at the point of application of the moment (crack mouth opening displacement) is computed as

36 $$\begin{aligned} \Delta =\Delta _{a}+\Delta _{CT}^{\varphi }+\Delta _{CT}^{\delta }, \end{aligned}$$

where

37 $$\begin{aligned} \Delta _{a}&= 2\frac{M\ {a}_0^2}{2\ EI}, \end{aligned}$$

38 $$\begin{aligned} \Delta _{CT}^{\varphi }&= -2\ \varphi _1(0)\ {a}_0=-2\left[ v_1^{\prime }(0)+\frac{{{\mathcal {T}}}_1(0)}{{\mu }{A}_s}\right] {a}_0\nonumber \\&=-2\ v_1^{\prime }(0)\ {a}_0, \end{aligned}$$

39 $$\begin{aligned} \Delta _{CT}^{\delta }&= 2\ v_1(0), \end{aligned}$$

are the crack mouth opening displacements due to bending of the arms behind the crack tip, rotation of the crack tip and opening at the crack tip, respectively. Note that displacement $\Delta$ is the total opening of the crack mouth, and it takes into account both arms, which is why factor 2 is used in expressions (37)–(39). Equation (36) can be now rewritten as

40 $$\begin{aligned} \Delta (M)=2\left[ \frac{M\ a_0^2}{2\ EI}-v_1^{\prime }(0)a_0+v_1(0)\right] , \quad M\le M_L, \nonumber \\ \end{aligned}$$

where $v_1(0)$ is defined in (34) and $v_1^{\prime }(0)$ can be obtained from (20) as

41 $$\begin{aligned} v_1^{\prime }(0)= & {} -\lambda (\zeta _1\ C_1+\zeta _2\ C_2)=\lambda (\zeta _4\ C_3-\zeta _3\ C_4)\nonumber \\= & {} -\dfrac{M\sqrt{2(1+\omega )}}{EI\ \lambda }, \end{aligned}$$

in which the final expression is valid for any value of $\omega$. It follows that

42 $$\begin{aligned}&\Delta (M)\nonumber \\&\quad =\frac{M\ {a}_0^2}{EI}\left[ 1+\frac{2\sqrt{2(1+\omega )}}{{a}_0\ \lambda }+\frac{2}{{a}_0^2 \lambda ^2}\right] , \quad M\le M_L,\nonumber \\ \end{aligned}$$

is obviously linear for any value of $\omega$ (including $\omega =1$, as reported in “Appendix A”).

We will use Eq. (36) as a general solution for the crack mouth opening displacement of a DCB, which is valid for all three solutions phases, not only for a DCB with prescribed rotations. However, in general $\Delta _a$, $\Delta _{CT}^{\varphi }$ and $\Delta _{CT}^{\delta }$ are computed differently each time.

The final forms of functions $v_1(x)$, $v_1^{\prime }(x)$, $\varphi _1(x)$, ${{\mathcal {M}}}_1(x)$, ${{\mathcal {T}}}_1(x)$ and $\sigma (x)$ for this phase are given in “Appendix E.1”.

### Phase of damage growth before crack propagation

In this phase, a cohesive (or damage-process) zone is developing in front of the crack tip. As already mentioned, for the cohesive zone ($x\in [-L_{cz},0]$) we use the solution (23), whereas for the zone of linear-elastic behaviour ($x\ge 0$) we use the solution (20).

There are six constants to determine, two for the undamaged part ($C_1$ and $C_2$ if $\omega >1$, or $C_3$ and $C_4$ if $\omega <1$) and four for the damaged part ($D_1,\ldots ,D_4$) in order to obtain the complete solution. First, from

43 $$\begin{aligned} v_1(0)=\frac{{\delta }_0}{2}, \end{aligned}$$

we obtain

44 $$\begin{aligned} C_1+C_2=C_4=\frac{{\delta }_0}{2}. \end{aligned}$$

We then impose the continuity conditions at the origin of the co-ordinate system:

45 $$\begin{aligned}&v_1(0)=v_2(0),\quad \varphi _1(0)=\varphi _2(0), \quad {{\mathcal {M}}}_1(0)={{\mathcal {M}}}_2(0),\nonumber \\&\quad {{\mathcal {T}}}_1(0)={{\mathcal {T}}}_2(0), \end{aligned}$$

where $\varphi _2(x)$ is the cross-sectional rotation on the damaged part of the interface, ${{\mathcal {M}}}_1(x)$ and ${{\mathcal {T}}}_1(x)$ are defined according to (26) and (27), respectively, and

46 $$\begin{aligned} {{\mathcal {M}}}_2(x)&= EI\ \varphi _2^{\prime }(x)\nonumber \\&=EI\left[ v_2^{\prime \prime }(x)+2\ \psi \ \kappa ^2\left( v_2(x)-\frac{{\delta }_c}{2}\right) \right] , \end{aligned}$$

47 $$\begin{aligned} {{\mathcal {T}}}_2(x)&= {{\mathcal {M}}}_2^{\prime }(x)=EI\left[ v_2^{\prime \prime \prime }(x)+2\ \psi \ \kappa ^2v_2^{\prime }(x)\right] , \end{aligned}$$

are the cross-sectional bending moment and the shear force on the damaged part of the interface, respectively. Using the solution (23) these expressions can be written as

48 $$\begin{aligned} {{\mathcal {M}}}_2(x)&= EI\ \kappa ^2\left\{ -\xi _1^2\left[ \sin (\kappa \ \xi _2\ x)D_1+\cos (\kappa \ \xi _2\ x)D_2\right] \right. \nonumber \\&\left. \quad +\,\xi _2^2\left[ \sinh (\kappa \ \xi _1\ x)D_3+\cosh (\kappa \ \xi _1\ x)D_4\right] \right\} , \end{aligned}$$

49 $$\begin{aligned} {{\mathcal {T}}}_2(x)&= EI\ \kappa ^3\left\{ -\xi _1\left[ \cos (\kappa \ \xi _2\ x)D_1-\sin (\kappa \ \xi _2\ x)D_2\right] \right. \nonumber \\&\left. \quad +\,\xi _2\left[ \cosh (\kappa \ \xi _1\ x)D_3+\sinh (\kappa \ \xi _1\ x)D_4\right] \right\} , \end{aligned}$$

where the property $\xi _1\ \xi _2=1$ is used to simplify the expressions. Note also that, because $\varphi _2(x)=v_2^{\prime }(x)+{{\mathcal {T}}}_2(x)/{\mu }{A}_s$ and ${{\mathcal {T}}}_1(0)={{\mathcal {T}}}_2(0)$, the condition $\varphi _1(0)=\varphi _2(0)$ can be written as $v_1^{\prime }(0)=v_2^{\prime }(0)$. From conditions (45) we can then express constants $D_i$ ($i=1,\ldots ,4$), in terms of $C_2$ for $\omega >1$ or $C_3$ for $\omega <1$ in the following general form

50 $$\begin{aligned} D_i(L_{cz})=\frac{D_{i1}+C_j(L_{cz})\ D_{i2}}{D_0}, \quad i=1,\ldots ,4, \end{aligned}$$

where $j=2$ for $\omega >1$ and $j=3$ for $\omega <1$, $D_{i1}$ and $D_{i2}$ are constants depending on the value of $\omega$, as explained below, and

51 $$\begin{aligned} D_0=2(\xi _1^2+\xi _2^2)\equiv 4(\psi ^2+1). \end{aligned}$$

As indicated in (50) and explained later, $D_i$ and $C_j$ are not true constants, but parameters depending on $L_{cz}$. If we set

52 $$\begin{aligned} {\overline{\zeta }}_1&= \left\{ \begin{array}{ll} \sqrt{\omega +\sqrt{\omega ^2-1}}, &{}\quad \text {if }\omega>1\\ 0, &{}\quad \text {otherwise} \end{array} \right. , \nonumber \\ {\overline{\zeta }}_2&= \left\{ \begin{array}{ll} \sqrt{\omega -\sqrt{\omega ^2-1}}, &{}\quad \text {if }\omega >1\\ 0, &{}\quad \text {otherwise} \end{array} \right. ,\nonumber \\ {\overline{\zeta }}_3&= \left\{ \begin{array}{ll} \sqrt{\dfrac{1+\omega }{2}}, &{}\quad \text {if }\omega<1\\ 0, &{}\quad \text {otherwise} \end{array} \right. , \nonumber \\ {\overline{\zeta }}_4&= \left\{ \begin{array}{ll} \sqrt{\dfrac{1-\omega }{2}}, &{}\quad \text {if }\omega <1\\ 0, &{}\quad \text {otherwise} \end{array} \right. , \end{aligned}$$

the values of the constants $D_{i1}$ and $D_{i2}$ ($i=1,\ldots ,4$) are the following:

53 $$\begin{aligned} D_{11}&= -{\delta }_0\ \eta \ \xi _1\left[ {\overline{\zeta }}_1(\xi _2^2+\eta ^2\ {\overline{\zeta }}_2^2)+{\overline{\zeta }}_3(\xi _2^2+\eta ^2)\right] ,\nonumber \\ D_{12}&= 2\ \eta \ \xi _1(\xi _2^2-\eta ^2)({\overline{\zeta }}_1-{\overline{\zeta }}_2+{\overline{\zeta }}_4), \nonumber \\ D_{21}&= {\delta }_0\ \eta ^2({\overline{\zeta }}_2^2+{\overline{\zeta }}_3^2-{\overline{\zeta }}_4^2-\eta ^2\ \xi _2^2), \nonumber \\ D_{22}&= 2\ \eta ^2({\overline{\zeta }}_1^2-{\overline{\zeta }}_2^2+2\ {\overline{\zeta }}_3\ {\overline{\zeta }}_4), \nonumber \\ D_{31}&= -{\delta }_0\ \eta \ \xi _2\left[ {\overline{\zeta }}_1(\xi _1^2-\eta ^2\ {\overline{\zeta }}_2^2)+{\overline{\zeta }}_3(\xi _1^2-\eta ^2)\right] , \nonumber \\ D_{32}&= 2\ \eta \ \xi _2(\xi _1^2+\eta ^2)({\overline{\zeta }}_1-{\overline{\zeta }}_2+{\overline{\zeta }}_4),\nonumber \\ D_{41}&= -{\delta }_0\ \eta ^2({\overline{\zeta }}_2^2+{\overline{\zeta }}_3^2-{\overline{\zeta }}_4^2+\eta ^2\ \xi _1^2), \nonumber \\ D_{42}&= -D_{22}, \end{aligned}$$

with $\eta =\lambda /\kappa$. Parameter $C_j(L_{cz})$ is determined from the last (sixth) remaining boundary condition

54 $$\begin{aligned} {{\mathcal {T}}}_2(-L_{cz})=0, \end{aligned}$$

as

55 $$\begin{aligned} C_j(L_{cz})=-\frac{\xi _1\left[ D_{21}\ s_2(L_{cz}) +D_{11}\ c_2(L_{cz})\right] +\xi _2\left[ D_{41}\ sh_1(L_{cz})-D_{31}\ ch_1(L_{cz})\right] }{\xi _1\left[ D_{22}\ s_2(L_{cz})+D_{12}\ c_2(L_{cz})\right] +\xi _2\left[ D_{42}\ sh_1(L_{cz})-D_{32}\ ch_1(L_{cz})\right] }, \end{aligned}$$

where $$j=2,3$$ and

56 $$\begin{aligned} s_2(L_{cz})=&\sin (L_{cz}\ \kappa \ \xi _2),&c_2(L_{cz})=&\cos (L_{cz}\ \kappa \ \xi _2),\nonumber \\ sh_1(L_{cz})=&\sinh (L_{cz}\ \kappa \ \xi _1),&ch_1(L_{cz})=&\cosh (L_{cz}\ \kappa \ \xi _1). \end{aligned}$$

To summarise, parameters $D_i(L_{cz})$ needed to define displacement field (23) within the damaged zone are determined from (50) for any value of $\omega$, where $C_j(L_{cz})$ is computed from (55). For $\omega >1$, $C_2(L_{cz})$ is defined according to (55) ($j=2$) and $C_1(L_{cz})$ is obtained from (44) as $C_1(L_{cz})={\delta }_0/2-C_2(L_{cz})$. For $\omega <1$, $C_3(L_{cz})$ is computed from (55) ($j=3$) and, according to (44), $C_4={\delta }_0/2$ for any value of $L_{cz}$.

The value of the applied moment $M$ is increasing during this phase and $L_{cz}$ is strictly increasing with increasing $M$. Hence, $M$ will also depend on $L_{cz}$. This function, $M(L_{cz})$, can be obtained from the following condition

57 $$\begin{aligned} M(L_{cz})={{\mathcal {M}}}_2(-L_{cz}), \end{aligned}$$

with ${{\mathcal {M}}}_2(-L_{cz})$ computed from (48). Obviously, $L_{cz}$ and $M(L_{cz})$ can increase only up to a certain limit after which the crack begins to propagate and we enter the third phase of the solution. The crack starts to propagate as soon as the relative opening at the crack tip reaches the critical value ${\delta }_c$ or

58 $$\begin{aligned} v_2(-L_{cz})=\frac{{\delta }_c}{2}. \end{aligned}$$

This condition represents a highly non-linear equation in terms of $L_{cz}$, which contains trigonometric and hyperbolic functions, and cannot be solved in a closed form. Thus, a numerical solver is needed and in the present work we use a simple Newton-Raphson iterative procedure (more information regarding the numerical solver is given in Sect. 7). We will denote the solution of (58) as ${\overline{L}}_{cz}$ and the maximum applied moment by ${M}_{max}=M({\overline{L}}_{cz})$.

According to (36), where $\Delta _a$ can be still defined according to (37), but now

59 $$\begin{aligned} \Delta _{CT}^{\varphi }&= -2\ v_2^{\prime }(-L_{cz})\ {a}_0, \end{aligned}$$

60 $$\begin{aligned} \Delta _{CT}^{\delta }&= 2\ v_2(-L_{cz}), \end{aligned}$$

the crack mouth opening can be computed as

61 $$\begin{aligned}&\Delta (L_{cz})=2\left[ \frac{M(L_{cz})\ {a}_0^2}{2\ EI} \right. \nonumber \\&\left. \quad -\,v_2^{\prime }(-L_{cz})\ {a}_0+v_2(-L_{cz})\right] , \quad L_{cz}\in [0,{\overline{L}}_{cz}], \end{aligned}$$

where $v_2(-L_{cz})$ and $v_2^{\prime }(-L_{cz})$ are evaluated from (23) at the co-ordinate $x=-L_{cz}$.

### Crack propagation phase

In the previous phase (damage growth before crack propagation) the applied moment is increasing from $M_L$ to $M({\overline{L}}_{cz})={M}_{max}$. Since for a DCB with prescribed rotations there is no shear force at the crack tip during all phases, it means that only the applied moment *M* (same at the crack tip as at the point of application) is responsible for crack propagation. Obviously, the crack will propagate when the applied moment reaches the value ${M}_{max}$ and this value will not change as the crack propagates. A constant value of ${M}_{max}$ during crack propagation implies that the boundary conditions at the crack tip remain constant and that $L_{cz}={\overline{L}}_{cz}$, during crack propagation. This kind of behaviour is known as ‘steady-state crack propagation’. Thus, unlike in the previous phase, in this phase $L_{cz}$ is not a variable.

The interface is again divided in two domains, the undamaged one ($x\ge 0$), where function $v_1(x)$ is defined according to (20), and the damaged one ($x\in [-{\overline{L}}_{cz},0]$), where function $v_2(x)$ is defined according to (23). Continuity conditions (45) still apply and constants $D_i$ are now obtained as

62 $$\begin{aligned} D_i=\frac{D_{i1}+C_j\ D_{i2}}{D_0},\quad i=1,\ldots ,4,\quad j=2,3, \end{aligned}$$

which is similar, but not equivalent to (50), because $D_i$ and $C_j$ are now true constants and not functions of $L_{cz}$. On the other hand, constants $D_0$, $D_{i1}$ and $D_{i2}$ are still defined according to (51) and (53). Constant $C_j$ is now determined from the condition

63 $$\begin{aligned} v_2(-{\overline{L}}_{cz})=\frac{{\delta }_c}{2} \end{aligned}$$

as

64 $$\begin{aligned} C_j=\frac{D_{11}\ {\overline{s}}_2-D_{21}\ {\overline{c}}_2+ D_{31}\ {\overline{sh}}_1-D_{41}\ {\overline{ch}}_1}{-D_{12}\ {\overline{s}}_2+D_{22}\ {\overline{c}}_2-D_{32}\ {\overline{sh}}_1+D_{42}\ {\overline{ch}}_1}, \end{aligned}$$

where $$j=2,3$$ and

65 $$\begin{aligned} {\overline{s}}_2&= s_2({\overline{L}}_{cz})=\sin ({\overline{L}}_{cz}\ \kappa \ \xi _2), \nonumber \\ {\overline{c}}_2&= c_2({\overline{L}}_{cz})=\cos ({\overline{L}}_{cz}\ \kappa \ \xi _2),\nonumber \\ {\overline{sh}}_1&= sh_1({\overline{L}}_{cz})=\sinh ({\overline{L}}_{cz}\ \kappa \ \xi _1), \nonumber \\ {\overline{ch}}_1&= ch_1({\overline{L}}_{cz})=\cosh ({\overline{L}}_{cz}\ \kappa \ \xi _1). \end{aligned}$$

To summarise, constants $D_i$ ($i=1,\ldots ,4$) are determined from (62) for any value of $\omega$. For $\omega >1$, $C_2=C_j$, where $C_j$ is defined in (64) and $C_1={\delta }_0/2-C_2$, according to (44). For $\omega <1$, $C_3=C_j$, where $C_j$ is defined in (64) and $C_4={\delta }_0/2$, according to (44).

Since in the crack propagation phase the applied moment remains constant, it can be computed from (57) as $M_{max}=M({\overline{L}}_{cz}$). Crack mouth opening can be then computed as

66 $$\begin{aligned} \Delta (a)= & {} 2\left[ \frac{{M}_{max}\ a^2}{2\ EI}-v_2^{\prime }(-{\overline{L}}_{cz})a\right. \nonumber \\&\left. +\,v_2(-{\overline{L}}_{cz})\right] , \quad a\ge a_0, \end{aligned}$$

where, theoretically, the value of *a* can go to infinity. Note also that $v_2(-{\overline{L}}_{cz})$ and $v_2^{\prime }(-{\overline{L}}_{cz})$ are constants and thus the function $\Delta (a)$ in this phase is quadratic. Because in this phase $M$ does not change, $\Delta$ does not depend on $M$ and we cannot define $\Delta (M)$.

## DCB with prescribed displacement

In this section we consider a DCB with prescribed displacement where, according to Fig. 1b, at the left-hand end the bottom arm is pinned, whereas the upper arm is pulled upwards. In order to prescribe a displacement $\Delta$ at the left-hand side of the upper arm, a vertical force $F$ must be applied at the same place and in the same direction. Thus, unlike in the case of a DCB with prescribed rotations, at the cracked portion of a DCB with prescribed displacement there is bending and shear in the arms, which will make the problem slightly more complex. Furthermore, because the crack propagation in the case of a DCB with prescribed displacement is not steady-state ($L_{cz}$ changes during crack propagation), the solution for the third phase (crack propagation) will be also more complex, compared to the case of DCB with a prescribed rotations where $L_{cz}={\overline{L}}_{cz}$ is constant during crack propagation. Each phase of the solution is explained in detail in following sections.

### Linear-elastic phase

In this phase the boundary conditions at the crack tip read

67 $$\begin{aligned} {{\mathcal {M}}}_1(0)=F\ {a}_0, \quad {{\mathcal {T}}}_1(0)=F, \end{aligned}$$

from which, using (26) and (27), constants

68 $$\begin{aligned} C_1=\frac{F(\zeta _1+{a}_0\ \lambda )}{EI\ \lambda ^3(1-\zeta _2^2)}, \quad C_2=\frac{F(\zeta _2+{a}_0\ \lambda )}{EI\ \lambda ^3(1-\zeta _1^2)}, \end{aligned}$$

for $$\omega >1$$ and

69 $$\begin{aligned} C_3= & {} -\frac{F(\zeta _3^2-\zeta _4^2+{a}_0\ \lambda \ \zeta _3)}{EI\ \lambda ^3\ \zeta _4}, \nonumber \\ C_4= & {} \frac{F({a}_0\ \lambda +2\ \zeta _3)}{EI\ \lambda ^3}, \end{aligned}$$

for $\omega <1$ are determined. The limit value of the force, $F_L$, at which damage starts to develop at the interface is obtained from condition (33), which gives

70 $$\begin{aligned} v_1(0)=\frac{F}{EI\ \lambda ^3}\left( {a}_0\ \lambda +\sqrt{2(\omega +1)}\right) =\frac{{\delta }_0}{2}, \end{aligned}$$

so that

71 $$\begin{aligned} F_L=\frac{EI\ {\delta }_0\ \lambda ^3}{2\left( {a}_0\ \lambda +\sqrt{2(1+\omega )}\right) }. \end{aligned}$$

Note that a real value of $F_L$ is obtained for any value of $\omega$ (including $\omega =1$, as reported in “Appendix A”). The same applies to $v_1(0)$ in (70).

The prescribed displacement is in this case equal to the crack mouth opening $\Delta$, which we define according to (36), where now we have

72 $$\begin{aligned} \Delta _{a}&= 2\left( \frac{F\ {a}_0^3}{3\ EI}+\frac{F\ {a}_0}{{\mu }{A}_s}\right) , \end{aligned}$$

73 $$\begin{aligned} \Delta _{CT}^{\varphi }&= -2\ \varphi _1(0)\ {a}_0=-2\ {a}_0\left[ v_1^{\prime }(0)+\frac{F}{{\mu }{A}_s}\right] , \end{aligned}$$

74 $$\begin{aligned} \Delta _{CT}^{\delta }&= 2\ v_1(0). \end{aligned}$$

The crack mouth opening displacement can be then written as

75 $$\begin{aligned} \Delta (F)=2\left[ \frac{F\ {a}_0^3}{3\ EI}-v_1^{\prime }(0)\ {a}_0+v_1(0)\right] ,\quad F\le F_L,\nonumber \\ \end{aligned}$$

where $v_1(0)$ is defined in (70) and

76 $$\begin{aligned} v_1^{\prime }(0)=-\frac{F}{EI\ \lambda ^2}\left( 2\ \omega +1+{a}_0\ \lambda \sqrt{2(1+\omega )}\right) . \end{aligned}$$

Note that although the terms responsible for shear deformations in (72) and (73) cancel out in (75), shear deformability is taken into account through $\omega$ in (70) and (76). Equation (76) is valid for any value of $\omega$ (including $\omega =1$, as reported in “Appendix A”). Equation (75) can be finally written as

77 $$\begin{aligned} \Delta (F)= & {} \frac{2\ F\ {a}_0^3}{3\ EI}\left\{ 1+\frac{3\sqrt{2(1+\omega )}}{({a}_0 \lambda )^3}\left[ \sqrt{2(1+\omega )}{a}_0\ \lambda \right. \right. \nonumber \\&\left. \left. +\,({a}_0\ \lambda )^2+1\right] \right\} ,\quad F\le F_L. \end{aligned}$$

The final forms of functions $v_1(x)$, $v_1^{\prime }(x)$, $\varphi _1(x)$, ${{\mathcal {M}}}_1(x)$, ${{\mathcal {T}}}_1(x)$ and $\sigma (x)$ for this phase are given in “Appendix E.2”.

### Phase of damage growth before crack propagation

In this phase, we divide the interface into the undamaged domain ($x\ge 0$) and the damaged domain ($x\in [-L_{cz}, 0]$), and continuity conditions (45) between the two still apply. Using these conditions and condition (43) we can again define parameters $D_i(L_{cz})$, where $i=1,\ldots ,4$, using (50) and constants $C_1$ and $C_4$ using (44). However, solution (55) for parameters $C_j(L_{cz})$, where $j=2,3$, is no longer valid because the boundary condition (54) in the case of prescribed displacement becomes

78 $$\begin{aligned} {{\mathcal {T}}}_2(-L_{cz})=F(L_{cz}), \end{aligned}$$

which cannot give us the solution for $C_j(L_{cz})$ because the function $F(L_{cz})$ is yet unknown. An additional boundary condition

79 $$\begin{aligned} {{\mathcal {M}}}_2(-L_{cz})=F(L_{cz})\ {a}_0, \end{aligned}$$

gives

80 $$\begin{aligned} F(L_{cz})=\frac{M(L_{cz})}{{a}_0}, \end{aligned}$$

where $M(L_{cz})$ is defined in (57). Now, using (80), from (78) it follows that

81 $$\begin{aligned}&C_j(L_{cz})\nonumber \\&\quad =\frac{\beta _1\ s_2(L_{cz})+\beta _2\ c_2(L_{cz})+\beta _3\ sh_1(L_{cz})+\beta _4\ ch_1(L_{cz})}{\beta _5\ s_2(L_{cz})+\beta _6\ c_2(L_{cz})+\beta _7\ sh_1(L_{cz})+\beta _8\ ch_1(L_{cz})}, \nonumber \\ \end{aligned}$$

where $$j=2,3$$ and

82 $$\begin{aligned} \beta _1&= \xi _1^2(D_{11}+{a}_0\ \kappa \ \xi _2\ D_{21})\nonumber \\ \beta _2&= \xi _1^2(-D_{21}+{a}_0\ \kappa \ \xi _2\ D_{11}),\nonumber \\ \beta _3&= \xi _2^2(-D_{31}+{a}_0\ \kappa \ \xi _1\ D_{41})\nonumber \\ \beta _4&= \xi _2^2(D_{41}-{a}_0\ \kappa \ \xi _1\ D_{31}),\nonumber \\ \beta _5&= -\xi _1^2(D_{12}+{a}_0\ \kappa \ \xi _2\ D_{22})\nonumber \\ \beta _6&= \xi _1^2(D_{22}-{a}_0\ \kappa \ \xi _2\ D_{12}),\nonumber \\ \beta _7&= \xi _2^2(D_{32}-{a}_0\ \kappa \ \xi _1\ D_{42})\nonumber \\ \beta _8&= \xi _2^2(-D_{42}+{a}_0\ \kappa \ \xi _1\ D_{32}). \end{aligned}$$

Let us recall that, because of definitions (52) and (53), solution (81) for $C_j(L_{cz})$ automatically returns the value of $C_2(L_{cz})$ for $\omega >1$ and $C_3(L_{cz})$ for $\omega <1$. Thus, in the case when $\omega >1$ we compute $C_2(L_{cz})$ from (81) and then $C_1(L_{cz})$ follows from (44) as $C_1(L_{cz})={\delta }_0/2-C_2(L_{cz})$. For $\omega <1$ we compute $C_3(L_{cz})$ from (81), whereas $C_4$ is defined in (44) as $C_4={\delta }_0/2$. Using solution (81), from (50) we can then obtain parameters $D_i(L_{cz})$, where $i=1,\ldots ,4$, which are needed to compute $M(L_{cz})$ (see (57)) and finally $F(L_{cz})$ according to (80).

As explained in Sect. 3.2, in this phase $L_{cz}$ grows from 0 to a value corresponding to the initiation of crack propagation, i.e. transition to the third phase. This is a maximum value for $L_{cz}$, because during crack propagation $L_{cz}$ decreases and asymptotically tends to a minimum value when $a\rightarrow \infty$, as is discussed in the next section. Therefore, this initial maximum value of $L_{cz}$ at the initiation of crack propagation will be denoted by $L_{cz}^{max}$. In order to obtain this value, the same approach as for a DCB with prescribed rotations is followed, i.e. condition (58) is imposed. This is again a highly non-linear equation in terms of $L_{cz}$, which is solved numerically (in our approach Newton-Raphson procedure is used).

The prescribed displacement in the second phase can be computed as

83 $$\begin{aligned} \Delta (L_{cz})= & {} 2\left[ \frac{F(L_{cz})\ {a}_0^3}{3\ EI}-v_2^{\prime }(-L_{cz})\ {a}_0 \right. \nonumber \\&\left. +\,v_2(-L_{cz})\right] , \quad L_{cz}\in [0,L_{cz}^{max}], \end{aligned}$$

where $v_2(-L_{cz})$ and $v_2^{\prime }(-L_{cz})$ are evaluated using (23) at the co-ordinate $x=-L_{cz}$.

### Crack propagation phase

As previously mentioned, in the case of a DCB with prescribed displacement, the cohesive zone length decreases during crack propagation from $L_{cz}^{max}$ asymptotically approaching a lower limit value $L_{cz}^{min}$. This means that the crack propagation is not steady state, but it approaches steady state for infinitely long cracks (Dimitri et al. 2017). Because $L_{cz}^{min}$ corresponds to a steady-state crack propagation, it must have the same value as ${\overline{L}}_{cz}$ found in the identical DCB loaded with prescribed rotations, where crack propagation is always steady state, i.e. $L_{cz}^{min}={\overline{L}}_{cz}$. Note that both $L_{cz}^{max}$ and $L_{cz}^{min}$ are obtained by numerically solving Eq. (58), where constants $D_i$ ($i=1,\ldots ,4$) in (23) in the former case are computed using (81) in (50), whereas in the latter case they are computed using (55) in (50).

Continuity conditions (45) and condition (43) are still valid in the third phase, which gives us solution (50). From the condition

84 $$\begin{aligned} v_2(-L_{cz})=\frac{{\delta }_c}{2}, \end{aligned}$$

we obtain

85 $$\begin{aligned} C_j(L_{cz})=\frac{D_{11}\ s_2(L_{cz})-D_{21}\ c_2(L_{cz})+ D_{31}\ sh_1(L_{cz})-D_{41}\ ch_1(L_{cz})}{-D_{12}\ s_2(L_{cz})+D_{22}\ c_2(L_{cz})-D_{32}\ sh_1(L_{cz})+D_{42}\ ch_1(L_{cz})}, \end{aligned}$$

where $j=2,3$. Note that this expression is different from (64) because here $L_{cz}$ is a variable.

Function $F(L_{cz})$ is obtained from conditions (78) and (49). Note that $a$ is also a function of $L_{cz}$, i.e. $a=a(L_{cz})$, and can be determined from the condition

86 $$\begin{aligned} {{\mathcal {M}}}_2(-L_{cz})=F(L_{cz})\ a(L_{cz}), \end{aligned}$$

which gives

87 $$\begin{aligned} a(L_{cz})=\frac{M(L_{cz})}{F(L_{cz})}, \end{aligned}$$

where $M(L_{cz})$ and $F(L_{cz})$ are defined according (57) and (78), respectively.

Prescribed displacement can be now expressed as a function of $L_{cz}$ as

88 $$\begin{aligned} \Delta (L_{cz})= & {} 2\left[ \frac{F(L_{cz})\ a(L_{cz})^3}{3\ EI}-v_2^{\prime }(-L_{cz})\ a(L_{cz}) \right. \nonumber \\&\left. +\,v_2(-L_{cz})\right] , \quad L_{cz}\in [L_{cz}^{min},L_{cz}^{max}], \end{aligned}$$

where $v_2(-L_{cz})$ and $v_2^{\prime }(-L_{cz})$ are evaluated using (23) at the co-ordinate $x=-L_{cz}$.

#### Remark 4.1

Solutions developed in Sects. 3 and 4 (based on Timoshenko beam theory) represent general solutions from which we can easily derive three other particular cases, for DCBs with either rotations or displacement prescribed:

1. Solutions for Euler–Bernoulli beam theory. These solutions are obtained by simply letting the shear modulus $\mu \rightarrow \infty$ and are presented in “Appendix B”.
2. Solutions for a linear-elastic interface with brittle failure. These solutions, presented in Sect. 5 for Timoshenko beam theory and in “Appendix C” for Euler–Bernoulli beam theory, are obtained by letting ${\delta }_c\rightarrow {\delta }_0$, which means that there is no damage before crack propagation, i.e. only the first and the third solution phases remain.
3. LEFM solutions. These solutions, given both for Timoshenko beam theory in Sect. 6 and Euler–Bernoulli beam theory in “Appendix C”, are obtained from the solutions for a linear-elastic CZM with brittle failure by letting ${\delta }_0\rightarrow 0$.

$$\square $$

## Analytical solutions for a DCB with linear-elastic interface with brittle failure (EBT)

Here we assume that the interface behaves as linear-elastic up to a certain point after which brittle failure (instantaneous loss of cohesion) occurs. In our model, this is achieved by setting ${\delta }_0={\delta }_c$, which removes the softening branch in $\sigma -\delta$ diagram shown in Fig. 2. This also means that the second part of the solution (damage growth before crack propagation) does not exist and that $L_{cz}=0$ during crack propagation.

Referring to Sect. 2.2, we can now rewrite Eq. (6) as

89 $$\begin{aligned} \sigma (x)=\left\{ \begin{array}{ll} \sigma _{max}\dfrac{\delta (x)}{{\delta }_0}, &{} \text {if }\delta (x)<{\delta }_0,\\ 0, &{} \text {otherwise}. \end{array} \right. \end{aligned}$$

Solutions (20) for $v_1(x)$, as well as definitions (9), (12) and (15) for $\lambda$, $\omega$ and $\zeta _i$ ($i=1,\ldots ,4$) are still valid. Solution (23) for $v_2(x)$ is no longer applicable because $L_{cz}=0$.

We will now derive the solution for a DCB with a brittle interface, first for a DCB with prescribed rotations and then for a DCB with prescribed displacement.

### DCB with prescribed rotations

The first (linear-elastic) phase of the solution presented in Sect. 3.1 applies completely to the case of linear-elastic interface with brittle failure. Thus, no modifications in the expressions presented in Sect. 3.1 are needed. Moreover, this phase can be now called ‘linear-elastic behaviour before crack propagation.’ The crack mouth opening displacement in the phase of crack propagation now reads

90 $$\begin{aligned} \Delta (a)=\frac{M_{max}\ a^2}{EI}\left[ 1+\frac{2\sqrt{2(1+\omega )}}{a\ \lambda } +\,\frac{2}{a^2 \lambda ^2}\right] , \quad a\ge a_0, \nonumber \\ \end{aligned}$$

where $M_{max}$ is the value of the applied moment when the crack starts to propagate corresponding to $M_L$ defined in (35).

Since for a linear-elastic interface with brittle failure the critical energy release rate, $G_c$, can be written as

91 $$\begin{aligned} G_c=\frac{\sigma _{max}\ {\delta }_0}{2}, \end{aligned}$$

we can rewrite Eq. (9)$_1$ as

92 $$\begin{aligned} \lambda =\root 4 \of {\frac{4\ b\ G_c}{EI\ {\delta }_0^2}}, \end{aligned}$$

and from (35) obtain

93 $$\begin{aligned} M_{max}=\sqrt{EI\ b\ G_c}, \end{aligned}$$

which is equivalent to the well-known formula

94 $$\begin{aligned} G_c=\frac{M_{max}^2}{b\ EI}, \end{aligned}$$

used to compute $G_c$ (or the critical value of the J integral, $J_c$) for a DCB with prescribed rotations (Rice 1968; Freiman et al. 1973; Suo et al. 1992; Sørensen et al. 1996). Note that ${M}_{max}$ and $G_c$ are independent of the beam theory used, i.e. the shear deformability of the arms does not influence their values.

### DCB with prescribed displacement

For a DCB with prescribed displacement the first part of the solution presented in Sect. 4.1 is entirely valid in the case when the interface is linear-elastic with brittle failure. The peak load at the point when the crack starts to propagate, $F_{max}$, corresponds to $F_L$ defined in (71), from where by substituting (9) and (92) it follows that

95 $$\begin{aligned} F_{max}=\frac{\sqrt{EI\ b\ G_c}}{{a}_0+\sqrt{\frac{2}{\lambda ^2}+\frac{EI}{{\mu }{A}_s}}}. \end{aligned}$$

For the crack propagation phase we have

96 $$\begin{aligned} \Delta (a)= & {} 2\frac{F(a)\ a^3}{3\ EI}\left\{ 1+\frac{3\sqrt{2(1+\omega )}}{(a\lambda )^3}\left[ \sqrt{2(1+\omega )}a\ \lambda \right. \right. \nonumber \\&\left. \left. +\,(a\ \lambda )^2+1\right] \right\} , \quad a\ge {a}_0, \end{aligned}$$

where

97 $$\begin{aligned} F(a)=\frac{\sqrt{EI\ b\ G_c}}{a+\sqrt{\frac{2}{\lambda ^2}+\frac{EI}{{\mu }{A}_s}}}\equiv \frac{\lambda \sqrt{EI\ b\ G_c}}{a\lambda +\sqrt{2(1+\omega )}}. \end{aligned}$$

#### Remark 5.1

Note that presented solutions in terms of the applied load and the crack mouth opening displacement of a DCB with either prescribed rotations or prescribed displacement for the case of a linear-elastic interface with a brittle failure (EBT) are valid for all values of $\omega$ ($\omega <1$, $\omega =1$ and $\omega >1$). $\square$

## LEFM solutions for a DCB with prescribed rotations and a DCB with prescribed displacement: enhanced simple beam theory(ESBT)

The presented model for a linear-elastic interface with a brittle crack still allows some opening at the interface in the linear-elastic range before crack starts to propagate ($\delta (x)<{\delta }_0$). If this initial linear-elastic behaviour is excluded from the model by letting ${\delta }_0\rightarrow 0$, while keeping $G_c$ constant (which also results in $\sigma _{max}\rightarrow \infty$), we obtain solutions equivalent to those given by linear-elastic fracture mechanics (LEFM). However, we will not a-priori assume that the DCB arms act as if they were clamped at the crack tip, which is usually done in the SBT approach. In this way we will show that in the limit case of LEFM the arms rotate at the crack tip and in front of it (even though their centre-lines there remain straight), which means that the clamped conditions at the crack tip cannot be obtained even for an infinitely stiff perfectly brittle interface. This is due exclusively to the shear deformability of the arms, which is accounted for in Timoshenko beam theory. For Euler–Bernoulli beam theory, the limit case of LEFM indeed corresponds to SBT and the arms do not rotate at the crack tip and in front of it. Thus, for our LEFM-limit solution for Timoshenko beam theory we will adopt the term ‘enhanced simple beam theory’ (ESBT). In the following subsections we will present only the final results for a DCB with prescribed rotations and a DCB with prescribed displacement, respectively, while the complete derivation is given in “Appendix E”.

### DCB with prescribed rotations

As shown in “Appendix E”, in the limit case of LEFM for any $x\ge 0$ we have

98 $$\begin{aligned} v_1^L(x)&= 0 \end{aligned}$$

99 $$\begin{aligned} v_1^{\prime L}(x)&=\frac{M}{\sqrt{EI\ {\mu }{A}_s}}({{\mathcal {H}}}(x)-1), \end{aligned}$$

100 $$\begin{aligned} \varphi _1^L(x)&= -\frac{M}{\sqrt{EI\ {\mu }{A}_s}}e^{-\sqrt{\frac{{\mu }{A}_s}{EI}}x}, \end{aligned}$$

101 $$\begin{aligned} \sigma ^L(x)&= -\dfrac{M\ {\mu }{A}_s}{b\ EI}e^{-\sqrt{\frac{{\mu }{A}_s}{EI}}x}+\frac{M}{b}\sqrt{\frac{{\mu }{A}_s}{EI}}{{\mathcal {D}}}(x), \end{aligned}$$

102 $$\begin{aligned} {{\mathcal {T}}}_1^L(x)&= -M\sqrt{\dfrac{{\mu }{A}_s}{EI}}\left[ e^{-\sqrt{\frac{{\mu }{A}_s}{EI}}x}+({{\mathcal {H}}}(x)-1)\right] , \end{aligned}$$

103 $$\begin{aligned} {{\mathcal {M}}}_1(x)&= Me^{-\sqrt{\frac{{\mu }{A}_s}{EI}}x}, \end{aligned}$$

where ${{\mathcal {D}}}(x)$ is the Dirac distribution centred at zero (which means that, rigorously speaking, the stress is not a ‘proper’ function but a generalised one), and ${{\mathcal {H}}}(x)$ is the Heaviside function defined by:

104 $$\begin{aligned} {{\mathcal {H}}}(x)=\left\{ \begin{array}{l l} 0 &{} \text {for } x\le 0,\\ 1 &{} \text {for } x>0. \end{array} \right. \end{aligned}$$

From the above expressions we can see that, although there are no relative displacements at the crack tip and in front of it ($v_1(x)=0$ for any $x\ge 0$), rotations at the crack tip and in front of it are still allowed to occur when Timoshenko beam theory is used. On the one hand, this result is expected, because it is intuitive that the independence of rotation and deflection in Timoshenko beam theory allows this theory to capture the deformation in front of the crack tip, which occurs also in the LEFM limit. This is in contrast with Euler–Bernoulli theory (see “Appendix E.3”), for which the absence of displacements also means a zero rotation and, ultimately, no deformation in front of the crack tip, but also with the widely used assumption, made in the SBT, that the arms of a DCB act as if they were clamped at the crack tip. In other words, to the best of the authors’ knowledge, the ability of Timoshenko’s beam theory to capture the crack tip rotation also in the LEFM limit has not been explored so far, although something similar was done in EBT for a linear elastic interface with finite stiffness and brittle failure (Kanninen 1973; Williams 1989). This is why we call our approach ESBT.

Moreover, because the DCB arms deform (rotate) in front of crack tip, contact tractions at the interface, as well as the shear forces and bending moments in the arms, appear in front of the crack tip, with an exponential decay as $x\rightarrow \infty$. We can notice that at the crack tip there is a jump in the shear force (from 0 to $-M\sqrt{{\mu }{A}_s/EI}$) which corresponds to a transition in the bending moment diagram from the constant value $M$ in the cracked portion of the arms to the function (103) in front of the crack tip. This implies that in the limit case of LEFM there is a concentrated transversal cohesive force exchanged at the crack tip, so that the interface stress is the sum of a compressive smooth part and the Dirac distribution centred at zero. Because at the crack tip the cross-sectional rotations of the arms must be continuous and there is a jump in the shear force in the arms, the function $v_1^{\prime }(x)$ is also discontinuous due to $\varphi _1(x)=v_1^{\prime }(x)+{{\mathcal {T}}}_1(x)/{\mu }{A}_s$.

Expressions (98)–(103) are valid only for the phase of linear-elastic behaviour before crack propagation. However, analogous expressions for the phase of crack propagation can be obtained simply by substituting $M$ with ${M}_{max}$. Finally, according to (40), we can express the crack mouth opening displacement as

105 $$\begin{aligned} \Delta (M)&= \frac{M\ {a}_0^2}{EI}\left( 1+\frac{2}{{a}_0}\sqrt{\frac{EI}{{\mu }{A}_s}}\right) , \nonumber \\&\quad \text {before crack propagation } (M\le M_{max}), \end{aligned}$$

106 $$\begin{aligned} \Delta (a)&= \frac{M_{max}\ a^2}{EI}\left( 1+\frac{2}{a}\sqrt{\frac{EI}{{\mu }{A}_s}}\right) , \nonumber \\&\quad \text {during crack propagation } (a\ge {a}_0), \end{aligned}$$

where ${M}_{max}$ is defined in (93) and the second term in the parentheses in both expressions represents the rotation of the arms at the crack tip.

### DCB with prescribed displacement

As shown in “Appendix E”, in the limit case of LEFM for any $x\ge 0$ we have

107 $$\begin{aligned} v_1^L(x)&= 0, \end{aligned}$$

108 $$\begin{aligned} v_1^{\prime L}(x)&= \left( \frac{F}{{\mu }{A}_s}+\frac{F\ {a}_0}{\sqrt{EI\ {\mu }{A}_s}}\right) ({{\mathcal {H}}}(x)-1) \end{aligned}$$

109 $$\begin{aligned} \varphi _1^L(x)&= -\frac{F\ {a}_0}{\sqrt{EI\ {\mu }{A}_s}}e^{-\sqrt{\frac{{\mu }{A}_s}{EI}}x} , \end{aligned}$$

110 $$\begin{aligned} \sigma ^L(x)&= -\dfrac{F\ {a}_0\ {\mu }{A}_s}{b\ EI}e^{-\sqrt{\frac{{\mu }{A}_s}{EI}}x}\nonumber \\&\quad +\frac{F}{b}\left( 1+{a}_0\sqrt{\frac{{\mu }{A}_s}{EI}}\right) {{\mathcal {D}}}(x), \end{aligned}$$

111 $$\begin{aligned} {{\mathcal {T}}}_1^L(x)&= -F\ {a}_0\sqrt{\dfrac{{\mu }{A}_s}{EI}}\left[ e^{-\sqrt{\frac{{\mu }{A}_s}{EI}}x}+({{\mathcal {H}}}(x)-1)\right] \nonumber \\&\quad -F({{\mathcal {H}}}(x)-1), \end{aligned}$$

112 $$\begin{aligned} {{\mathcal {M}}}_1^L(x)&= F\ {a}_0\ e^{-\sqrt{\frac{{\mu }{A}_s}{EI}}x}, \end{aligned}$$

where again we see that even in the limit case of LEFM the arms rotate at and in front of the crack tip when Timoshenko beam theory is used. The discussion regarding Eqs. (98)–(103) also applies here, with the only difference that for a DCB with prescribed displacement in the cracked portion of the arms we have a constant shear force and a linear distribution of bending moments.

Note that expressions (107)–(112) are valid only for the phase of linear-elastic behaviour before crack propagation. However, analogous expressions for the crack propagation phase can be obtained by substituting $F$ with $F(a)$ (defined in (116)) and ${a}_0$ with $a$.

The crack mouth opening displacement before crack propagation follows from (75) as

113 $$\begin{aligned} \Delta (F)= & {} \frac{2\ F\ {a}_0^3}{3\ EI}\left( 1+\frac{3}{{a}_0^2}\frac{EI}{{\mu }{A}_s} \right. \nonumber \\&\left. +\,\frac{3}{{a}_0}\sqrt{\frac{EI}{{\mu }{A}_s}}\right) , \quad F\le F_{max}, \end{aligned}$$

where from (95) we have

114 $$\begin{aligned} F_{max}=\frac{\sqrt{EI\ b\ G_c}}{{a}_0+\sqrt{\frac{EI}{{\mu }{A}_s}}}. \end{aligned}$$

During crack propagation the crack mouth opening displacement is given by

115 $$\begin{aligned} \Delta (a)= & {} \frac{2\ F(a)\ a^3}{3\ EI}\left( 1+\frac{3}{a^2}\frac{EI}{{\mu }{A}_s}\right. \nonumber \\&\left. +\,\frac{3}{a}\sqrt{\frac{EI}{{\mu }{A}_s}}\right) , \quad a\ge {a}_0, \end{aligned}$$

where from (97) we have

116 $$\begin{aligned} F(a)=\frac{\sqrt{EI\ b\ G_c}}{a+\sqrt{\frac{EI}{{\mu }{A}_s}}}. \end{aligned}$$

Note that in (113) and (115) the term outside the parentheses represents the arm deflection due to bending according to Euler–Bernoulli beam theory, the second term in the parentheses is due to shear deformability of the arm, while the third term in the parentheses represents the influence of the rotation of the arms at the crack tip. From (116) we can obtain the critical energy release rate of a Timoshenko DCB with prescribed displacement as

117 $$\begin{aligned} G_c=\frac{F^2}{b}\left( \frac{a^2}{EI}+\frac{1}{{\mu }{A}_s}+\frac{2\ a}{\sqrt{EI\ {\mu }{A}_s}}\right) , \end{aligned}$$

where, for the sake of simplicity, we denote $F(a)$ simply by $F$. Usually in the SBT solution (Ripling et al. 1971; ASTM D3433-99 2012; BS ISO 25217:2009 2009), only the first two terms in expression (117) are taken into account because it is assumed that the DCB arms are clamped at the crack tip. The third therm in (117), which takes into account the rotation at the crack tip, to the best of authors’ knowledge, has not been recognised so far for the limit case of LEFM. Equation (117) represents the ESBT expression for $G_c$.

#### Remark 6.1

In “Appendix E.3” we show that if Euler–Bernoulli beam theory is used, there are no cross-sectional rotations, shear forces or bending moments of the arms in front of the crack tip. However, singularity of contact tractions at the interface, as well as discontinuity of the shear stresses and bending moments in the arms, take place at the crack tip. These conditions are equivalent to clamping DCB arms at the crack tip and explain why the formulae obtained for the limit case of LEFM in “Appendix D” indeed correspond to widely used formulae in SBT. $\square$

## Numerical examples

In this section, for a DCB with a bi-linear CZM at the interface, the analytical solutions derived in this paper using Timoshenko beam theory will be compared to the numerical results obtained with an equivalent finite-element (FE) model, in which the same beam theory and CZM are used (Škec et al. 2015), and to the Euler–Bernoulli beam theory analytical solutions derived in “Appendix B”. The latter will allow us to investigate the influence of shear deformability of DCB arms on the results. LEFM solutions obtained in Sect. 6 will also be presented as limit cases for a brittle interface.

Referring to Fig. 1, we consider a DCB with dimensions $h=6$ mm, $b=25$ mm and ${a}_0=30$ mm. In the present analytical solution, the length of the specimen is assumed to be infinite ($L=\infty$), i.e. the crack is always sufficiently distant from the right-hand (non-loaded) end of the DCB. Material data for the DCB arms and the interface used in numerical examples is presented in Table 1, where it can be noted that the maximum contact traction $\sigma _{max}$ is varied between 7.5 and 120 MPa, while keeping the area under the traction–separation law, $\Omega$, constant. This gives us 5 cases of different brittleness of the interface, where $\sigma _{max}=7.5$ MPa represents an extremely ductile case and $\sigma _{max}=120$ MPa an extremely brittle case. Ratio $\alpha ={\delta }_0/{\delta }_c$ is kept constant in the first set of examples ($\alpha =0.01$ for all cases), whereas in the last example is varied. Values of $E$ and $\nu$ for the DCB arms presented in Table 1 correspond to aluminum, the shear modulus $\mu$ is calculated as for an isotropic material, i.e. $\mu =0.5E/(1+\nu )$, and for the rectangular cross-section considered, $k_s=5/6$.

**Table 1:** Material data used in the numerical examples. Except in the last example, $\alpha =0.01$

*E* (GPa)	$\nu$ (–)	$k_s$ (–)	$\Omega$ (N/mm)	$\sigma _{max}$ (MPa)	${\delta }_c$ (mm)	${\delta }_0$ (mm)
70	1/3	5/6	1	$\{7.5, 15, 30, 60, 120\}$	$2\ \Omega /\sigma _{max}$	$\alpha \ {\delta }_c$

**Fig. 3:**
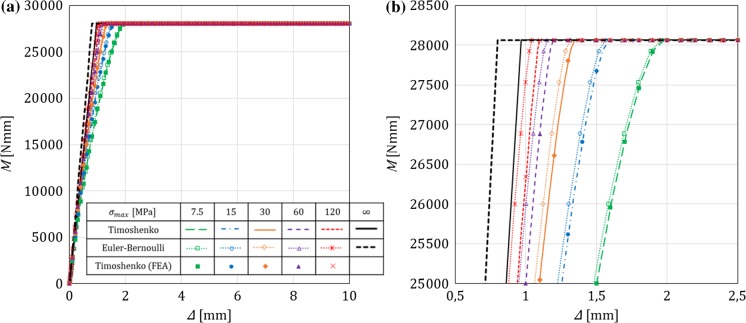
Crack mouth opening displacement–applied moment ($\Delta {-}M$) graph for a DCB with prescribed rotations: **a** range of interest, **b** zoom. A comparison between Timoshenko and Euler–Bernoulli beam theory for different values of $\sigma _{max}$, where $\sigma _{max}=\infty$ represents the LEFM solution

The numerical model used is the multi-layer beam model presented in Škec et al. (2015) where we assume a total length of the specimen $L=200$ mm. A total number of 2000 2-node Timoshenko beam elements are distributed evenly over the upper half of the DCB, meaning that the element length is 0.1 mm. Such a fine mesh is used to eliminate or at least minimise the influence of discretisation-caused spurious oscillations on the results (Alfano and Crisfield 2001; Škec et al. 2015). A 4-node interface element is attached to every beam FE from $x={a}_0$ to $x=L$ making a total of 1700 interface elements. The solution is obtained using displacement control and Newton-Raphson iterative procedure. Because our numerical model has 4002 degrees of freedom (one transverse displacement and one cross-sectional rotation per node), in each iteration of each increment, 4002 linear equations are solved in order to obtain the cross-head displacement. In our analytical solution, we obtain the cross-head displacement from a single closed-form solution. The same applies to any other quantity we want to obtain. Furthermore, all analytical solutions, unlike the numerical ones, are perfectly smooth.

It is worth noting that the values reported in Table 1 according to (9)$_2$ for $\sigma _{max}=\{7.5,15,30,60,120\}$ MPa give $\omega =\{0.32,0.64,1.28,2.57,5.13\}$. Thus, we can deduce that in real-life applications both $\omega <1$ and $\omega >1$ are possible and therefore the analytical solution should account for both cases.

In the following sections we will present the results for the DCB first with prescribed rotations and then with prescribed displacement.

### DCB with prescribed rotations

In Fig. 3, the reaction moment, $M$, is plotted against the crack mouth opening displacement, $\Delta$. From Fig. 3b, which is the zoom of Fig. 3a, we can first observe that the results for Timoshenko beam theory from the analytical and the FE model perfectly match, as expected. We can also note that the results approach the LEFM solution, which is obtained from our model by letting ${\delta }_0\rightarrow 0$ and $\sigma _{max}\rightarrow \infty$ (as shown in Sect. 5.1), as $\sigma _{max}$ increases. The same behaviour can be observed when Euler–Bernoulli beam theory is used. Differences between Timoshenko and Euler–Bernoulli beam theories are more pronounced for higher values of $\sigma _{max}$ and especially for the LEFM solution.

**Fig. 4:**
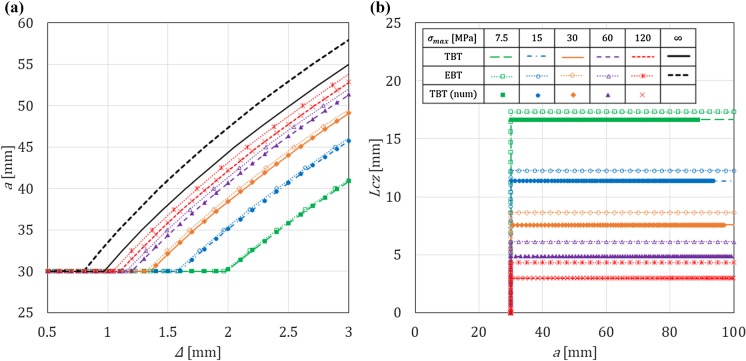
**a** Crack mouth opening displacement–crack length ($\Delta {-}a$) graph and **b** crack length–cohesive zone length ($a{-}L_{cz}$) graph for a DCB with prescribed rotations. A comparison between Timoshenko and Euler–Bernoulli beam theory for different values of $\sigma _{max}$, where $\sigma _{max}=\infty$ represents the LEFM solution

From Fig. 4a it can be clearly seen that the crack will start to propagate sooner (i.e. for smaller crack mouth opening displacements) when the interface is more brittle. The numerical model again agrees perfectly with the analytical solution, and there are some differences between Euler–Bernoulli and Timoshenko beam theory, which again become more significant as $\sigma _{max}$ increases.

In Fig. 4b we can finally compare the cohesive zone lengths, $L_{cz}$, for Timoshenko and Euler–Bernoulli beam theories. As expected, $L_{cz}$, which is highly influenced by the value of $\sigma _{max}$, remains constant during crack propagation. Differences between Timoshenko and Euler–Bernoulli beam theory solutions are now even more pronounced, especially for more brittle cases. Again, the numerical results match perfectly with those obtained from the analytical solution for Timoshenko beam theory.

### DCB with prescribed displacement

In this section, the same geometrical and material data as in Sect. 7.1 is used for the case of a DCB with prescribed displacement. We present a comparison of the solution from Sect. 4 for Timoshenko beam theory with the analogous solution for Euler–Bernoulli beam theory (presented in “Appendix B.2”) and the LEFM solution. This is done by comparing the values of the applied force, $F$, computed for each model at the same value of the crack mouth opening displacement, $\Delta$. Recall that in Sects. 4.2 and 4.3, in the solution phases which include damage, we have defined all quantities as functions of $L_{cz}$. Therefore, once the limit values $L_{cz}^{max}$ and $L_{cz}^{min}$ have been obtained numerically, a series of suitable values of $L_{cz}$ can be chosen for the second and the third phase and the solution of the problem is obtained analytically in a closed form. On the other hand, for the purpose of the present rigorous numerical comparison, it is necessary to express these quantities in terms of $\Delta$. However, deriving functions $F(\Delta )$, $a(\Delta )$ and $L_{cz}(\Delta )$ from expressions given Sects. 4.2 and 4.3 is impossible, because the functional dependence on $L_{cz}$ is highly non-linear. Thus, values of $F$, $a$ and $L_{cz}$ for a certain value of $\Delta$ are determined numerically using Newton–Raphson procedure. This computational effort is still negligible in comparison with the standard FEA because we only need to solve a single non-linear equation for each increment of $\Delta$.

**Fig. 5:**
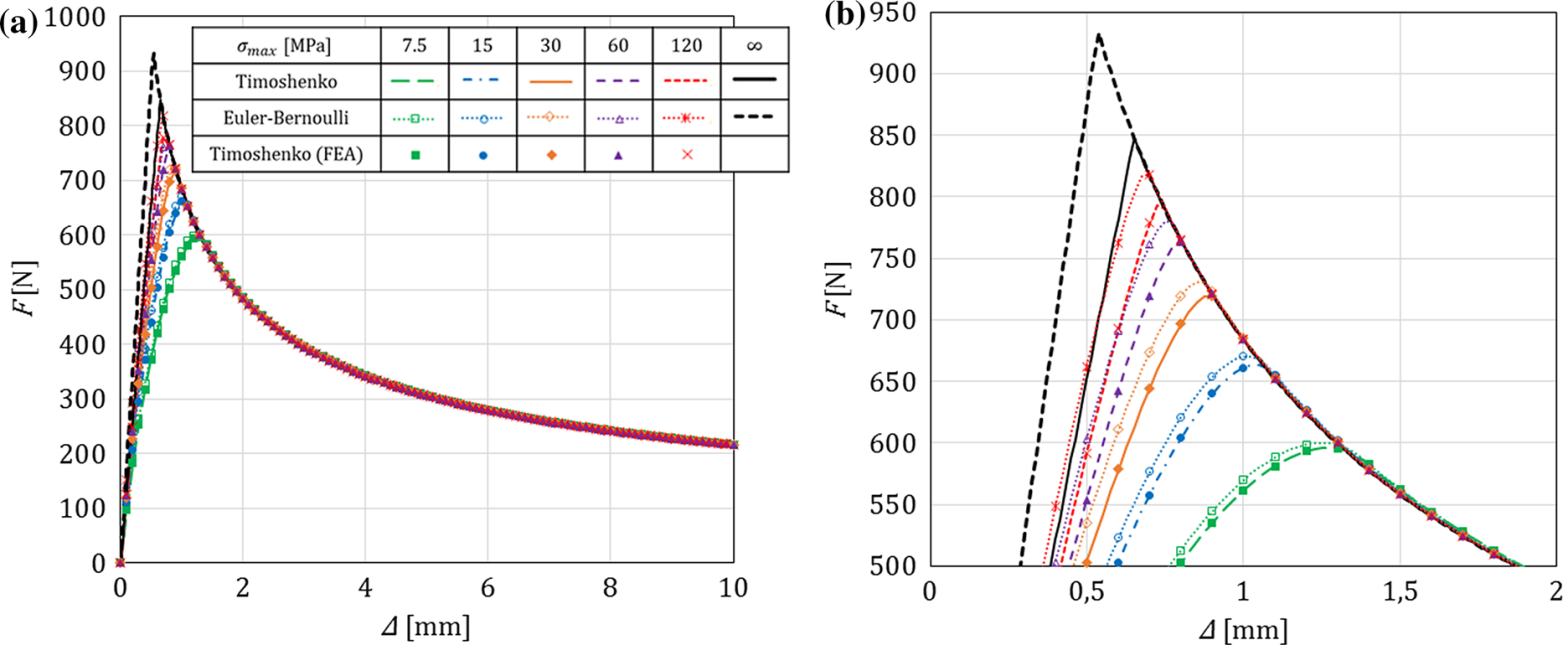
Crack mouth opening displacement–applied force ($\Delta {-}F$) graph for a DCB with prescribed displacement: **a** range of interest, **b** zoom. A comparison between Timoshenko and Euler–Bernoulli beam theory for different values of $\sigma _{max}$, where $\sigma _{max}=\infty$ represents the LEFM solution

**Fig. 6:**
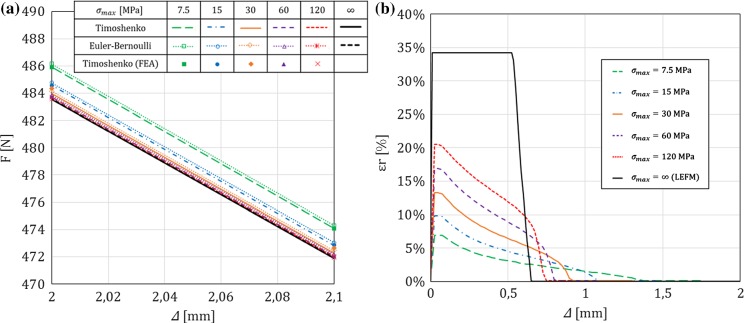
Comparison of **a**$\Delta {-}F$ data during crack propagation (a closer look) and **b** relative errors when using Euler–Bernoulli instead of Timoshenko beam theory for different values of $\sigma _{max}$

In Fig. 5, the reaction force, $F$, is plotted against the prescribed crack mouth opening displacement, $\Delta$, for different values of $\sigma _{max}$. Both Timoshenko and Euler–Bernoulli beam theories are used. We can see that $\sigma _{max}$ has a considerable influence on the results in the first two phases (before crack starts to propagate), especially in the second phase, when the stiffness of the DCB progressively decreases as damage is developing in front of the crack tip. This also results in a reduction of the peak force. In Fig. 5b it can again be seen that differences between Timoshenko and Euler–Bernoulli beam theory become more pronounced as $\sigma _{max}$ increases, i.e. the interface becomes more brittle. Results from the FE model based on the Timoshenko beam theory perfectly match the results obtained from the analytical solution. In the third phase (crack propagation) all curves are extremely close, but they do not coincide perfectly. This is better shown in Fig. 6a, where it can be noted that the differences of the results in the crack propagation phase indeed exist, but they are too small to be appreciated on a normal scale.

In fact, a simple argument explains why the curve corresponding to a finite value of $\sigma _{max}$ must lie above the LEFM solution after a point which coincides with the start of crack propagation. For finite values of $\sigma _{max}$, the gradual development of the cohesive zone ahead of the initial crack tip, before the crack starts propagating, is responsible for the nonlinear deviation of the load-displacement curve from the initial straight line of the LEFM solution, culminating in the rounded part so that, the lower $\sigma _{max}$, the lesser the peak load with respect to the theoretical peak load predicted by LEFM. In terms of energy, this means that, in the case of a finite value of $\sigma _{max}$, before the crack starts propagating, less external work is performed by the external force, $F$, than for the LEFM case. However, once the DCB is completely separated, the total amount of external work must be equal to the interface area times the area under the traction–separation law, that is the work of separation, $\Omega$. Therefore, for a DCB with an infinite length, the only way that the total external work spent is the same for the two cases is that, for a finite value of $\sigma _{max}$, the curve lies above the case for $\sigma _{max}\rightarrow \infty$ during crack propagation, so that the increase in external work in this part of the curve compensates the lower amount of external work before crack propagation.

We can define a relative error due to using Euler–Bernoulli instead of Timoshenko beam theory as

118 $$\begin{aligned} \varepsilon _r=\frac{\vert F_{T}-F_{E}\vert }{F_{T}}, \end{aligned}$$

where, for the same value of $\sigma _{max}$, $F_T$ and $F_E$ are the values of the applied force $F$ at the same $\Delta$ computed using Timoshenko and Euler–Bernoulli beam theory, respectively. By comparing Fig. 6b with Fig. 5b, we can see that this error has the highest values in the first (linear-elastic) phase. During the second phase the relative error decreases, and the higher the strength $\sigma _{max}$ the more rapid the reduction in the error. In the third phase, the error is negligible.

**Fig. 7:**
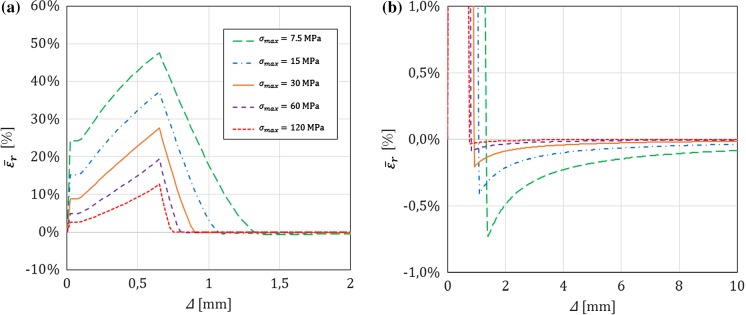
Timoshenko beam theory. Relative differences between the values of the force $F$ computed for a finite value of $\sigma _{max}$ and for $\sigma _{max}=\infty$ (LEFM solution): **a** crack initiation, **b** crack propagation

**Fig. 8:**
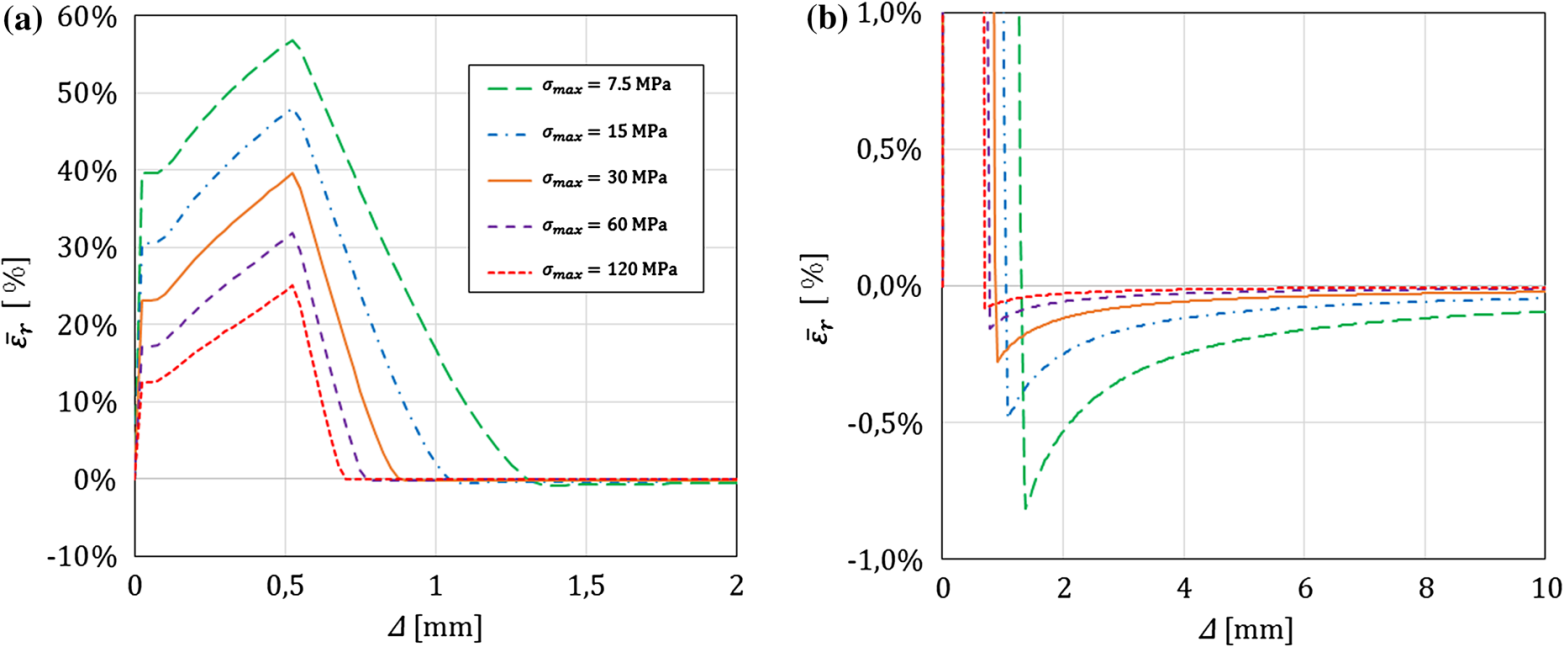
Euler–Bernoulli beam theory. Relative differences between the values of the force $F$ computed for a finite value of $\sigma _{max}$ and for $\sigma _{max}=\infty$ (LEFM solution): **a** crack initiation, **b** crack propagation

We can also compare the relative differences in the results with respect to LEFM solutions by introducing factor ${\overline{\varepsilon }}_r$ defined by:

119 $$\begin{aligned} {\overline{\varepsilon }}_r=\frac{\mid F_{\infty }-F_{\sigma _{max}}\mid }{F_{\infty }}, \end{aligned}$$

where $F_{\sigma _{max}}$ and $F_{\infty }$ are the values of the applied force $F$ computed for the same crack mouth opening displacement for different values of $\sigma _{max}$ and for $\sigma _{max}=\infty$, respectively. We can compute ${\overline{\varepsilon }}_r$ separately for Timoshenko and Euler–Bernoulli beam theory. These results are presented in Fig. 7 for Timoshenko beam theory and Fig. 8 for Euler–Bernoulli beam theory. As expected, the highest values of the relative differences are obtained for more ductile interfaces (with smaller $\sigma _{max}$). However, the most important result is that these relative differences are extremely small during the crack propagation phase for both beam theories. This means that the most important parameter in the CZM, $\Omega$, can be computed accurately enough using $F-\Delta$ data in the crack propagation phase and the simple LEFM formula (D.7), derived in “Appendix D” and based on Euler–Bernoulli beam theory assuming that $\Omega =G_c$. This is because we have already demonstrated that the differences in the $F-\Delta$ curves computed with Timoshenko and Euler–Bernoulli beam theories are negligible in the crack propagation phase (see Fig. 6b) for the case under examination. However, we have to keep in mind that for anisotropic materials (such as composites), where the shear modulus can be significantly smaller than Young’s modulus, the differences between the results obtained with Timoshenko and Euler–Bernoulli beam theory could be more pronounced.

In Fig. 5 we noted that $\sigma _{max}$ significantly influences the peak load reached before the crack starts to propagate and dictates how far from brittle behaviour (LEFM) the considered CZM solution is. However, changing the ratio, $\alpha$, between ${\delta }_0$ and ${\delta }_c$ has a noticeable influence on the results, too. We will assume that $\alpha$ can vary between a value very close to 0 (meaning that ${\delta }_0$ is almost negligible compared to ${\delta }_c$) and 1 (meaning that ${\delta }_0={\delta }_c$). The latter case, which is covered in Sect. 5.2, implies that we have a linear-elastic behaviour at the interface up to ${\delta }_0$ (or ${\delta }_c$) followed by brittle failure (leading to $L_{cz}=0$).

**Fig. 9:**
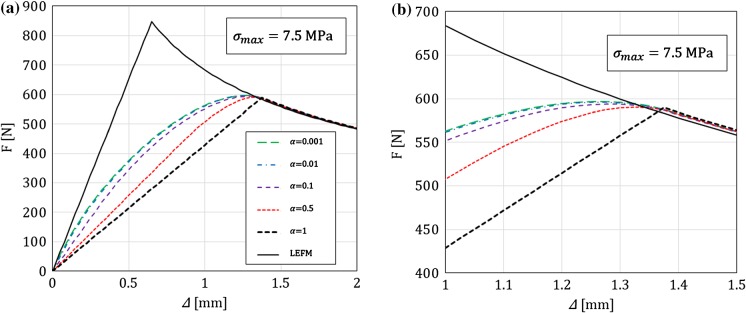
The influence of $\alpha ={\delta }_0/{\delta }_c$ on the $\Delta -F$ results for $\sigma _{max}=7.5$ MPa: **a** range of interest, **b** a closer look

**Fig. 10:**
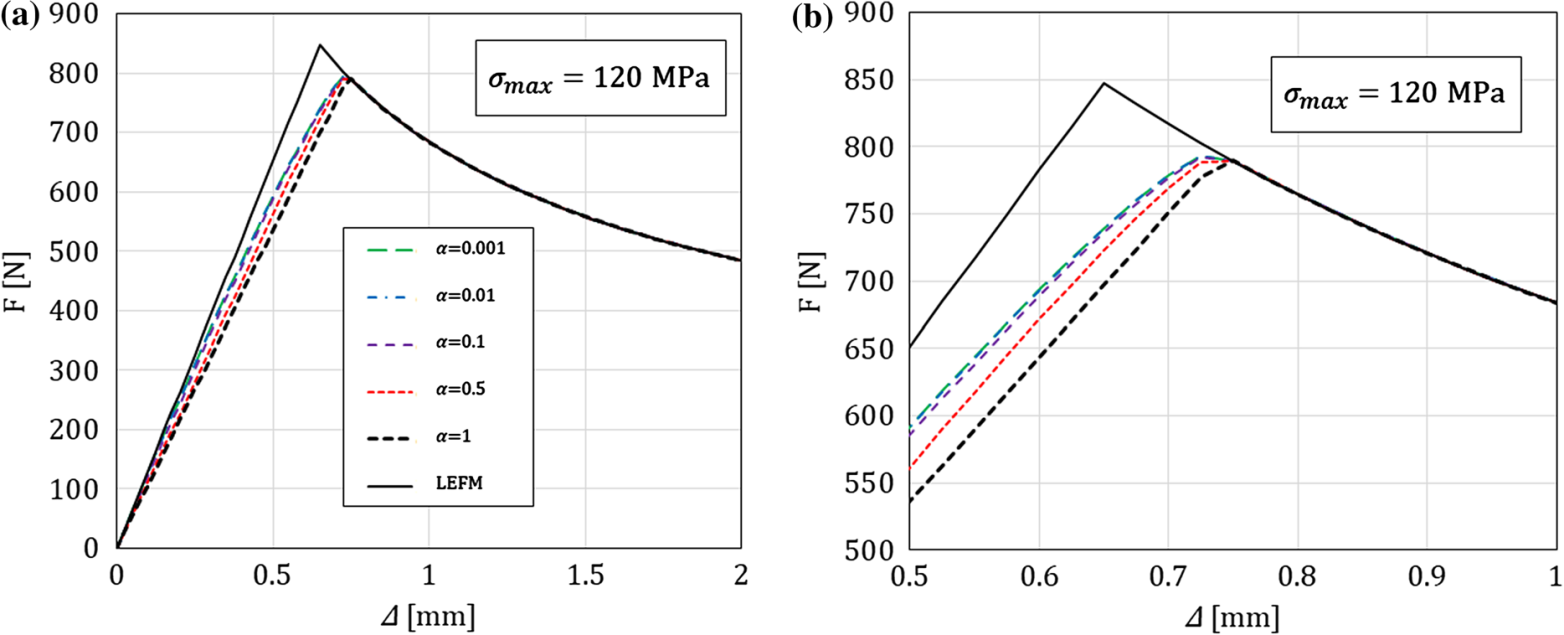
The influence of $\alpha ={\delta }_0/{\delta }_c$ on the $\Delta -F$ results for $\sigma _{max}=120$ MPa: **a** range of interest, **b** a closer look

In Figs. 9 and 10 we can see that increasing $\alpha$ reduces the stiffness of the interface. For $\alpha =1$ the phase of damage growth before the crack tip does not exist and the behaviour before crack propagation is linear-elastic. We can see that the influence of $\alpha$ on the results is more pronounced for $\sigma _{max}=7.5$ MPa than for $\sigma _{max}=120$ MPa, because, for the same $\Omega$, smaller $\sigma _{max}$ results in larger ${\delta }_c$. It can be noted, however, that $\alpha$ does not significantly influence the value of the peak force, especially for the brittle case.

### Behaviour in front of the crack tip for a DCB with linear-elastic interface with brittle crack and in the limit case of LEFM

In Sect. 6 (see also “Appendix E”) we showed that in the limit case of LEFM stresses and strains are found in front of the crack tip when Timoshenko beam theory is used to model the arms. In “Appendix E.3” we show that this is not the case when Euler–Bernoulli beam theory is used to model the arms. In this section, using the same geometrical and material data for the bulk material as in the previous examples, we will show that the behaviour of a DCB with a linear-elastic interface with brittle crack approaches the behaviour described in Sect. 6 for the limit case of LEFM as the stiffness of the interface increases. We will consider only the case of a DCB with prescribed displacement and investigate the case when the crack starts to propagate, which means that in Eqs. (107)–(112) we use $F(a)$ (defined in (116)) and $a$ instead of $F$ and ${a}_0$. However, in this example we will assume that $a={a}_0=30$ mm, which means that we will investigate the case when the crack starts to propagate from its initial position. Note that, because the crack propagation for a DCB with prescribed displacement is not steady state, the presented results would change for $a>{a}_0$, eventually approaching the steady-state solutions for $a\rightarrow \infty$. These solutions are given by Eqs. (98)–(103) for a DCB with prescribed rotations (where ${M}_{max}$ should be used instead of $M$ for the crack propagation phase).

In this example we are again assuming that $\Omega =1$ N/mm, where $\Omega =\sigma _{max}\ {\delta }_0/2$. The values of $\sigma _{max}$ are varied according to $\sigma _{max}=10^i$ MPa, where $i=0,1,2,3,4$, and the values of ${\delta }_0$ follow from ${\delta }_0=2\ \Omega /\sigma _{max}$.

**Fig. 11:**
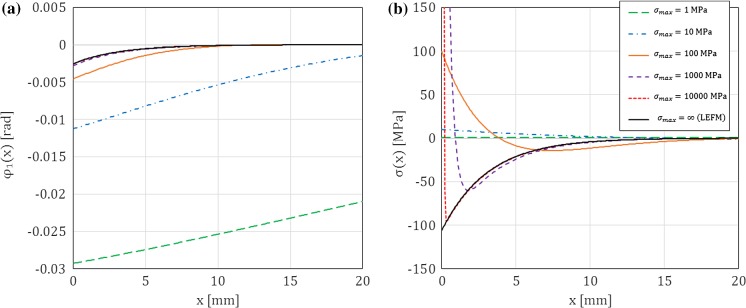
Distribution of: **a** cross-section rotations of the upper arm, $\varphi _1(x)$ and **b** contact tractions at the interface, $\sigma (x)$, in front the crack tip for a DCB with prescribed displacement and a linear-elastic interface with a brittle crack for different values of $\sigma _{max}$. The presented results correspond to the start of the crack propagation from the initial crack ($a={a}_0=30$ mm)

In Fig. 11a we see that the cross-sectional rotations of the upper arm in front of the crack tip, $\varphi _1(x)$, reduce as the interface becomes stiffer, but for the limit case of LEFM, instead of approaching zero, they approach the limit values given by function (109). According to this formula, the rotation at the crack tip for the limit case of LEFM is $\varphi (0)=-\,0.0025$ rad. In Fig. 11b we see how the contact tractions at the crack tip ($\sigma (0)=\sigma _{max}$) increase for higher values of $\sigma _{max}$ and tends to $\infty$ in the LEFM limit. In front of the crack tip, there is a distribution of compressive stresses which in the limit case of LEFM converges to the curve given by Eq. (110) according to which $\lim _{x\rightarrow 0^+}\sigma (x)=-\,105.98$ MPa. This shows that the area of tensile stresses reduces to zero, while the stress at $x=0$ tends to infinity. It is shown in “Appendix E” that the resultant of these tensile stresses tends to a finite value. In other words, in the LEFM limit, a Dirac distribution centred at the crack tip is found.

**Fig. 12:**
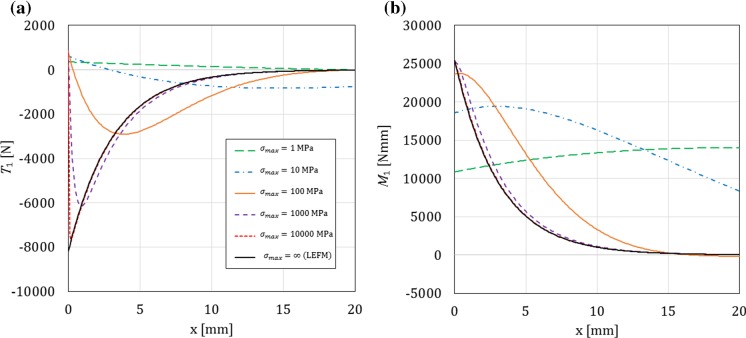
Distribution of: **a** shear forces, ${{\mathcal {T}}}_1(x)$ and **b** bending moments, ${{\mathcal {M}}}_1(x)$, in the upper arm in front of the crack tip for a DCB with prescribed displacement and a linear-elastic interface with a brittle crack for different values of $\sigma _{max}$. The presented results correspond to the start of the crack propagation from the initial position ($a={a}_0=30$ mm)

Figure 12 shows the distribution of shear forces in the upper arm in front of the crack tip, ${{\mathcal {T}}}_1(x)$. We can see that for higher values of the stiffness the force rapidly drops from the positive value ${{\mathcal {T}}}_1(0)=F(a)$ to a negative value. In the limit case of LEFM, according to (111), a discontinuity is found between $\lim _{x\rightarrow 0^-}{{\mathcal {T}}}_1(x)=F(a)$ and $\lim _{x\rightarrow 0^+}{{\mathcal {T}}}_1(x)=-8209.26$ N. In Fig. 12b, the distribution of bending moments in the upper arm in front of the crack tip, ${{\mathcal {M}}}_1(x)$, is shown. At the crack tip ${{\mathcal {M}}}_1(x)=F(0) a$, which for the limit case of LEFM, according to (112), is ${{\mathcal {M}}}_1(0)=25435.47$ Nmm.

It is worth noting that using Euler–Bernoulli beam theory in the limit case of LEFM we have $\varphi _1(x)=\sigma (x)={{\mathcal {T}}}_1(x)={{\mathcal {M}}}_1(x)=0$ for $x>0$, but $\varphi _1(0)=0$, $\sigma (0)=\infty$, ${{\mathcal {T}}}_1(0)=F(a)$ and ${{\mathcal {M}}}_1(0)=F(a)a$ at the crack tip ($x=0$).

## Conclusions

In this paper we have derived complete analytical solutions for DCB specimens where the arms are modelled using simple beam theories (Timoshenko or Euler–Bernoulli). At the interface, three different models have been assumed: (i) a quasi-brittle bi-linear CZM, (ii) a liner-elastic CZM with brittle failure and (iii) a perfectly brittle and infinitely stiff interface (corresponding to LEFM solutions). The models obtained for each mentioned type of interface are called ‘cohesive crack model’ (CCM), ‘enhanced beam theory’ (EBT) and ‘enhanced simple beam theory’ (ESBT), respectively. In our approach EBT solutions are obtained from CCM solutions by removing the softening branch (responsible for progressive damage) from the CZM. From there, ESBT solutions can be obtained by letting the interface stiffness go to infinity. We have introduced a new term ESBT because we show that in the limit case of LEFM, EBT model does not correspond to ‘standard beam theory’ (SBT) model where the DCB arms act as if they were clamped at the crack tip. In ESBT, the arms are allowed to rotate at and in front of the crack tip, which is due exclusively to the shear deformability of the arms. This is why for the case when the arms are not shear-deformable (Euler–Bernoulli beam theory), ESBT corresponds to SBT. We have also derived the CCM, EBT and SBT solutions for the case when Euler–Bernoulli beam theory is used to model the arms by simply letting the shear stiffness of the arms go to infinity. All the mentioned solutions are derived for two different types of DCB: one with prescribed rotations and one with prescribed displacement. The presented solutions allow us to easily compute the crack mouth opening displacement, applied load (moment or force), contact tractions at the interface, displacement and rotations of the arms ahead of the crack tip, and shear forces and bending moments in the arms for different DCB models.

The main novel contributions of the paper are as follows:

1. The complete analytical solutions given in a unified and compact form for DCBs (either with prescribed rotations or prescribed displacement) with a bi-linear CZM at the interface.
2. The complete analytical solutions given in a unified and compact form for DCBs (either with prescribed rotations or prescribed displacement) with linear-elastic interface with brittle failure. It is shown that such EBT solutions, compared to CMM solutions, are very accurate in the phase of crack propagation. However, they are not able to capture the quasi-brittle behaviour before crack propagation.
3. The solutions for the limit case of LEFM which, in the case of Timoshenko beam theory (ESBT), show that at and in front of the crack tip the arms are allowed to rotate even if the interface is infinitely stiff. This implies that Timoshenko beam theory is, in a way, capable of capturing the realistic behaviour of the DCB in front of the crack tip and that the widely used assumption that in LEFM the arms act as if they were clamped at the crack is not necessary. However, we show that this assumption is still valid when the arms are modelled as Euler–Bernoulli beams.
4. An expression for the critical energy release rate, $G_c$, for a DCB with prescribed displacement is proposed, which, to the best of authors’ knowledge, is original. This expression, compared to the formula derived under the assumption that the arms are clamped at the crack tip, has an additional term which is dependent on the shear deformability of the arms and accounts for the rotations of the arms at the crack tip. If the arms are non-deformable in shear (Euler–Bernoulli beam theory), the expression for $G_c$ corresponds to the one obtained under the assumptions that the arms are clamped at the crack tip.

Future work will include the assessment of the accuracy of the formula for $G_c$ which in LEFM limit case takes into account the rotations of the arms at the crack tip. Similar analyses have been already done by Biel and Stigh (2008) and the authors of the present work (Škec et al. 2018). Based on the data obtained from the experiments or more sophisticated numerical models, our intention is to compare the accuracy of this formula with formulae from BS ISO 25217:2009 (2009).

It is also worth noting that, using the approach presented in this paper, obtaining the analytical solution for a DCB with trapezoidal CZM at the interface using the Timoshenko beam theory to model the arms should be straight-forward and will also be covered in future work.

## Free software made available

All the results are implemented in a software application with a user-friendly graphic interface where Euler–Bernoulli or Timoshenko beam theory for the arms and CCM, EBT or ESBT models for the interface can be selected. Results of the analysis can be plotted and exported. Because the computations are based on the presented analytical solutions, the results in our software are obtained instantaneously, even on a regular laptop computer. The software is free and can be downloaded at http://dx.doi.org/10.17633/rd.brunel.7223795.

## Supplementary data

Supplementary material related to this article can be found online at http://dx.doi.org/10.17633/rd.brunel.7212218.

## Appendix A: Solutions for Timoshenko beam theory when $\omega =1$

### Appendix A.1: DCB with prescribed rotations

In the phase of linear-elastic behaviour, Eqs. (24) and (25) become

A.1 $$\begin{aligned} {{\mathcal {M}}}_1(x)&=EI\left[ v_1^{\prime \prime }(x)-2\ \lambda ^2\ v_1(x)\right] , \end{aligned}$$

A.2 $$\begin{aligned} {{\mathcal {T}}}_1(x)&=EI\left[ v_1^{\prime \prime \prime }(x)-2\ \lambda ^2\ v_1^{\prime }(x)\right] , \end{aligned}$$

which, after substituting (20) for the case when $\omega =1$, gives

A.3 $$\begin{aligned} {{\mathcal {M}}}_1(x)&= -EI\ \lambda ^2\ e^{-\lambda x}\left[ C_5+C_6\left( x+\frac{2}{\lambda }\right) \right] , \end{aligned}$$

A.4 $$\begin{aligned} {{\mathcal {T}}}_1(x)&= EI\ \lambda ^3\ e^{-\lambda x}\left[ C_5+C_6\left( x+\frac{1}{\lambda }\right) \right] . \end{aligned}$$

From here, using the boundary conditions (30) we obtain

A.5 $$\begin{aligned} C_5=\frac{M}{EI\ \lambda ^2}, \quad C_6=-\frac{M}{EI\ \lambda }. \end{aligned}$$

From boundary condition (33) it follows that

A.6 $$\begin{aligned} v_1(0)=C_5=\frac{M}{EI\ \lambda ^2}, \end{aligned}$$

which is the same result obtained in (34) for $\omega >1$ and $\omega <1$. This means that the moment $M_L$ can be expressed using (35) for any value of $\omega$ (including $\omega =1$). Because

A.7 $$\begin{aligned} v_1^{\prime }(0)=C_6-\lambda \ C_5=-\dfrac{2\ M}{EI\ \lambda }, \end{aligned}$$

is equivalent to setting $\omega =1$ in (41), Eq. (42) is valid for any value of $\omega$, and for $\omega =1$ it can be written as

A.8 $$\begin{aligned} \Delta (M)=\frac{M\ {a}_0^2}{EI}\left[ 1+\frac{4}{{a}_0\ \lambda }+\frac{2}{({a}_0\ \lambda )^2}\right] , \quad M\le M_L. \end{aligned}$$

In the phase of damage growth before crack propagation, from boundary condition (43) we obtain

A.9 $$\begin{aligned} C_5=\frac{{\delta }_c}{2}. \end{aligned}$$

From the continuity conditions (45) we obtain

A.10 $$\begin{aligned} D_i(L_{cz})=\frac{D_{i1}+C_6(L_{cz})\ D_{i2}}{D_0},\quad i=1,\ldots ,4, \end{aligned}$$

where the constants $D_{i1}$ and $D_{i2}$ read

A.11 $$\begin{aligned} D_{11}&= -{\delta }_0\ \eta \ \xi _1(\xi _2^2+\eta ^2),&D_{12}&= 2\frac{\xi _1}{\kappa } (\xi _2^2-\eta ^2), \nonumber \\ D_{21}&= {\delta }_0\ \eta ^2(1-\eta ^2\ \xi _2^2),&D_{22}&= 4\frac{\eta }{\kappa }, \nonumber \\ D_{31}&= -{\delta }_0\ \eta \ \xi _2(\xi _1^2-\eta ^2),&D_{32}&= 2\frac{\xi _2}{\kappa }(\xi _1^2+\eta ^2),\nonumber \\ D_{41}&= -{\delta }_0\ \eta ^2(1+\eta ^2\ \xi _1^2),&D_{42}&= -D_{22}. \end{aligned}$$

Parameter $C_6(L_{cz})$ is defined as for $C_j(L_{cz})$ in (55). Expressions (57) for $M(L_{cz})$ and (61) for $\Delta (L_{cz})$ are valid also for $\omega =1$.

In the phase of crack propagation, constant $C_5$ is still obtained from (A.9) and constants $D_i$ from the expression analogous to (62), i.e.

A.12 $$\begin{aligned} D_i=\frac{D_{i1}+C_6\ D_{i2}}{D_0},\quad i=1,\ldots ,4, \end{aligned}$$

where $C_6$ is defined as for $C_j$ in (64). The crack mouth opening displacement $\Delta (a)$ is computed according to (66).

### Appendix A.2: DCB with prescribed displacement

Using expressions (A.1) and (A.2), from boundary conditions (67) we obtain

A.13 $$\begin{aligned} C_5=\frac{F({a}_0\ \lambda +2)}{EI\ \lambda ^3}, \quad C_6=-\frac{F({a}_0\ \lambda +1)}{EI\ \lambda ^2}. \end{aligned}$$

From boundary condition (33) we obtain

A.14 $$\begin{aligned} v_1(0)=C_5=\frac{F({a}_0\ \lambda +2)}{EI\ \lambda ^3}, \end{aligned}$$

which gives as the limit value of the force

A.15 $$\begin{aligned} F_L=\frac{EI\ {\delta }_0\ \lambda ^3}{2({a}_0\ \lambda +2)}. \end{aligned}$$

The latter result implies that Eq. (71) is valid also for the case when $\omega =1$. Furthermore, (A.14) and

A.16 $$\begin{aligned} v_1^{\prime }(0)=-\frac{F(2\ {a}_0\ \lambda +3)}{EI\ \lambda ^2} \end{aligned}$$

show that expressions (70) and (76) are valid also for $\omega =1$. Thus, the crack mouth opening can be computed according to (77) as

A.17 $$\begin{aligned} \Delta (F)=\frac{2\ F\ {a}_0^3}{3\ EI}\left[ 1+\frac{6\left( {a}_0\ \lambda +1\right) ^2}{({a}_0 \lambda )^3}\right] ,\quad F\le F_L. \end{aligned}$$

For the second phase of the solution we again obtain (A.9) and (A.10) from condition (43) and the continuity conditions (45), respectively. Constant $C_6(L_{cz})$ is defined as for $C_j(L_{cz})$ in (81), where the definitions (A.11) for constants $D_{i1}$ and $D_{i2}$ ($i=1,\ldots ,4$) must be used. We can express $F(L_{cz})$ using (80) and $\Delta (L_{cz})$ using (83).

For the phase of crack propagation we compute $D_i$ ($i=1,\ldots ,4$) according to (A.12), where $C_6(L_{cz})$ is computed as $C_j(L_{cz})$ in (85) with definitions (A.11) for constants $D_{i1}$ and $D_{i2}$ ($i=1,\ldots ,4$). Constant $C_5$ is still defined according to (A.9). Functions $F(L_{cz})$ and $a(L_{cz})$ are computed from (78) and (87), respectively. Finally, $\Delta (L_{cz})$ can be computed from (88).

## Appendix B: Solutions for Euler–Bernoulli beam theory

In this section, we will assume that DCB arms are shear-undeformable by letting $\mu \rightarrow \infty$. This will simply transform solutions for Timoshenko beam theory presented in Sects. 3 and 4 into solutions for Euler–Bernoulli beam theory.

According to (9)$_2$ and (10)$_2$, in the limit of $\mu \rightarrow \infty$ both $\omega$ and $\psi$ tend to 0. This means that on the undamaged part of the interface the solution will be defined as for the case when $\omega <1$ in Sect. 2.2.1. Setting $\omega =\psi =0$, from (15) and (22), respectively, we now obtain

B.1 $$\begin{aligned} \zeta _3=\zeta _4=\frac{\sqrt{2}}{2}, \quad \text {and} \quad \xi _1=\xi _2=1. \end{aligned}$$

The displacements of the upper arm on the undamaged and damaged part of the interface read

B.2 $$\begin{aligned} v_1(x)&= e^{-\lambda _E\ x}\left[ \sin (\lambda _E\ x)C_3 \right. \nonumber \\&\quad \left. +\cos (\lambda _E\ x)C_4\right] , \quad \text {for } x\ge 0, \end{aligned}$$

B.3 $$\begin{aligned} v_2(x)&= \sin (\kappa \ x)D_1+\cos (\kappa \ x)D_2+\sinh (\kappa \ x)D_3\nonumber \\&\quad +\cosh (\kappa \ x)D_4 +\frac{{\delta }_c}{2}, \quad \text {for } x\in [-L_{cz},0], \end{aligned}$$

respectively, where $\lambda _E=\sqrt{2}\lambda /2$. Definitions (B.2) and (B.3) are now used to solve the problems of a DCB with either rotations or displacement prescribed.

### Appendix B.1: DCB with prescribed rotations

The three-phase solution procedure reported in Sect. 3 is followed. For the liner-elastic phase we have

B.4 $$\begin{aligned} C_3=-C_4, \quad C_4=\frac{M}{EI\lambda ^2}=\frac{M}{2\ EI\ \lambda _E^2} \end{aligned}$$

and

B.5 $$\begin{aligned} M_L=\frac{EI\ {\delta }_0\ \lambda ^2}{2}=EI\ {\delta }_0\ \lambda _E^2. \end{aligned}$$

The crack mouth opening displacement in the linear-elastic phase can be expressed using (42) as

B.6 $$\begin{aligned} \Delta (M)=\frac{M\ {a}_0^2}{EI}\left( 1+\frac{1}{{a}_0\ \lambda _E}\right) ^2, \quad M\le M_L. \end{aligned}$$

For the phase of damage growth before crack propagation, we use (50) to determine constants $D_i$ ($i=1,\ldots ,4$), where now $C_j(L_{cz})=C_3(L_{cz})$. From (44) we know that

B.7 $$\begin{aligned} C_4=\frac{{\delta }_0}{2}. \end{aligned}$$

By taking into account (B.1), from (51) we get

B.8 $$\begin{aligned} D_0=4, \end{aligned}$$

and from (53)

B.9 $$\begin{aligned} D_{11}=&-{\delta }_0\ \eta _E(1+2\ \eta _E^2),&D_{12}=&2\ \eta _E(1-2\ \eta _E^2),\nonumber \\ D_{21}=&-4\ {\delta }_0\ \eta _E^4,&D_{22}=&4\ \eta _E^2,\nonumber \\ D_{31}=&-{\delta }_0\ \eta _E(1-2\ \eta _E^2),&D_{32}=&2\ \eta _E(1+2\ \eta _E^2),\nonumber \\ D_{41}=&D_{21},&D_{42}=&-D_{22}, \end{aligned}$$

where $\eta _E=\lambda _E/\kappa$. Constant $C_3(L_{cz})$ is expressed from (55) for $j=3$ and it reads

B.10 $$\begin{aligned} C_3(L_{cz})=-\frac{D_{21}\sin (\kappa \ L_{cz})+D_{11}\cos (\kappa \ L_{cz})+D_{41}\sinh (\kappa \ L_{cz})-D_{31}\cosh (\kappa \ L_{cz})}{D_{22}\sin (\kappa \ L_{cz})+D_{12}\cos (\kappa \ L_{cz})+D_{42}\sinh (\kappa \ L_{cz})-D_{32}\cosh (\kappa \ L_{cz})}. \end{aligned}$$

Equations (57) and (48) give the solution for $M(L_{cz})$ which is then used to numerically compute the value of ${\overline{L}}_{cz}$ from Eq. (58) so that $M_{max}=M({\overline{L}}_{cz})$. The crack mouth opening displacement in the second phase is computed from (61), where $v_2(L_{cz})$ and $v_2^{\prime }(L_{cz})$ are obtained from (B.3).

In the third phase we obtain constants $D_i$ ($i=1,\ldots ,4$) from (62) by using expressions (B.8) and (B.9) for $D_0$, $D_{i1}$ and $D_{i2}$ and assuming that now $C_j=C_3$. Equation (64) now becomes

B.11 $$\begin{aligned} C_3=\frac{D_{11}\ \sin (\kappa \ {\overline{L}}_{cz})-D_{21} \cos (\kappa \ {\overline{L}}_{cz})+ D_{31}\ \sinh (\kappa \ {\overline{L}}_{cz})-D_{41}\ \cosh (\kappa \ {\overline{L}}_{cz})}{-D_{12}\ \sin (\kappa \ {\overline{L}}_{cz})+D_{22}\ \cos (\kappa \ {\overline{L}}_{cz})-D_{32}\ \sinh (\kappa \ {\overline{L}}_{cz})+D_{42}\ \cosh (\kappa \ {\overline{L}}_{cz})}. \end{aligned}$$

The crack mouth opening displacement in the phase of crack propagation is computed according to (66).

### Appendix B.2: DCB with prescribed displacement

In the linear-elastic phase constants (69) for the solution (B.2) become

B.12 $$\begin{aligned} C_3=-\frac{F\ {a}_0}{2\ EI\ \lambda _E^2}, \quad C_4=\frac{F({a}_0\ \lambda _E+1)}{2\ EI\lambda _E^3}. \end{aligned}$$

From (71) we obtain

B.13 $$\begin{aligned} F_L=\frac{EI\ {\delta }_0\ \lambda _E^3}{1+{a}_0\ \lambda _E}. \end{aligned}$$

The crack mouth opening displacement is obtained from (77) as

B.14 $$\begin{aligned} \Delta (F)&= 2\frac{F\ {a}_0^3}{3\ EI}\left\{ 1+\frac{3}{2({a}_0 \lambda _E)^3}\left[ 2\ {a}_0\ \lambda _E\right. \right. \nonumber \\&\quad \left. \left. +\,2({a}_0\ \lambda _E)^2+1\right] \right\} ,\quad F\le F_L. \end{aligned}$$

In the second phase, we define $D_i(L_{cz})$ ($i=1,\ldots ,4$) according to (50), where we use (B.8) and (B.9) and assume that $C_j(L_{cz})=C_3(L_{cz})$. The latter is defined according to (81) as

B.15 $$\begin{aligned} C_3(L_{cz})=\frac{\beta _1\ \sin (\kappa \ L_{cz})+\beta _2\ \cos (\kappa \ L_{cz})+\beta _3\ \sinh (\kappa \ L_{cz})+\beta _4\ \cosh (\kappa \ L_{cz})}{\beta _5\ \sin (\kappa \ L_{cz})+\beta _6\ \cos (\kappa \ L_{cz})+\beta _7\ \sinh (\kappa \ L_{cz})+\beta _8\ \cosh (\kappa \ L_{cz})}, \end{aligned}$$

where

B.16 $$\begin{aligned} \beta _1&= D_{11}+{a}_0\ \kappa \ D_{21},&\beta _2&= -D_{21}+{a}_0\ \kappa \ D_{11},\nonumber \\ \beta _3&= -D_{31}+{a}_0\ \kappa \ D_{41},&\beta _4&= D_{41}-{a}_0\ \kappa \ D_{31},\nonumber \\ \beta _5&= -D_{12}-{a}_0\ \kappa \ D_{22},&\beta _6&= D_{22}-{a}_0\ \kappa \ D_{12},\nonumber \\ \beta _7&= D_{32}-{a}_0\ \kappa \ D_{42},&\beta _8&= -D_{42}+{a}_0\ \kappa \ D_{32}, \end{aligned}$$

with constants $D_{i1}$ and $D_{i2}$ ($i=1,\ldots ,4$) defined in (B.9). $F(L_{cz})$ is computed according to (80), where $M(L_{cz})$ is defined in (57). The limit value $L_{cz}^{max}$ is determined from Eq. (58) which is solved numerically. The crack mouth opening displacement is computed from (83).

In the third phase we use (85) to express $C_3(L_{cz})$ as

B.17 $$\begin{aligned} C_3(L_{cz})=\frac{D_{11}\ \sin (\kappa \ L_{cz})-D_{21}\ \cos (\kappa \ L_{cz})+ D_{31}\ \sinh (\kappa \ L_{cz})-D_{41}\ \cosh (\kappa \ L_{cz})}{-D_{12}\ \sin (\kappa \ L_{cz})+D_{22}\ \cos (\kappa \ L_{cz})-D_{32}\ \sinh (\kappa \ L_{cz})+D_{42}\ \cosh (\kappa \ L_{cz})}, \end{aligned}$$

where $D_{i1}$ and $D_{i2}$ ($i=1,\ldots ,4$) are defined in (B.9). Function $F(L_{cz})$ is obtained from condition (78) and (49), whereas $a(L_{cz})$ is expressed using (87). Finally, the crack mouth opening displacement for the third phase can be computed according to (88).

## Appendix C: Euler–Bernoulli beam theory solutions for a DCB with linear-elastic interface with brittle failure (EBT)

### Appendix C.1: DCB with prescribed rotations

In the linear-elastic phase before crack propagation we compute the crack mouth opening displacement according to (B.6), whereas for the crack propagation phase we have

C.1 $$\begin{aligned} \Delta (a)=\frac{M_{max}\ a^2}{EI}\left( 1+\frac{1}{a\ \lambda _E}\right) ^2,\quad a\ge {a}_0, \end{aligned}$$

where $M_{max}$ is defined in (93).

### Appendix C.2: DCB with prescribed displacement

The crack mouth opening displacement in the linear-elastic phase before crack propagation is computed according to (B.14). The peak load, as defined in (95), now becomes

C.2 $$\begin{aligned} F_{max}=\frac{\sqrt{EI\ b\ G_c}}{{a}_0+\frac{1}{\lambda _E}}. \end{aligned}$$

In the crack propagation phase, the crack mouth opening displacement is computed as

C.3 $$\begin{aligned} \Delta (a)= & {} 2\frac{F(a)\ a^3}{3\ EI}\left\{ 1+\frac{3}{2(a\ \lambda _E)^3}\left[ 2\ a\ \lambda _E \right. \right. \nonumber \\&\quad \left. \left. +2(a\ \lambda _E)^2+1\right] \right\} ,\quad a\ge {a}_0, \end{aligned}$$

where according to (97) we now have

C.4 $$\begin{aligned} F(a)=\frac{\sqrt{EI\ b\ G_c}}{a+\frac{1}{\lambda _E}}. \end{aligned}$$

## Appendix D: LEFM solutions for a DCB using Euler–Bernoulli beam theory (SBT)

### Appendix D.1: DCB with prescribed rotations

Letting $\mu \rightarrow \infty$ in (105) and (106), or $\lambda _E\rightarrow \infty$ in (B.6) and (C.1), results in the following expressions for the crack mouth opening displacement when Euler–Bernoulli beam theory is used:

D.1 $$\begin{aligned} \Delta (M)&= \frac{M\ {a}_0^2}{EI}, \nonumber \\&\quad \text {before crack propagation } (M\le M_{max}),\end{aligned}$$

D.2 $$\begin{aligned} \Delta (a)&= \frac{M_{max}\ a^2}{EI}, \nonumber \\&\quad \text {during crack propagation } (a\ge {a}_0), \end{aligned}$$

where $M_{max}$ is defined in (93). In contrast to formulae (105) and (106), where Timoshenko beam theory is used, from these expressions, it is obvious that for Euler–Bernoulli beam theory the arms of a DCB in the limit case of LEFM indeed act as if they were clamped at the crack tip.

### Appendix D.2: DCB with prescribed displacement

By letting $\mu \rightarrow \infty$ in (113) and (115), or $\lambda _E\rightarrow \infty$ in (B.14) and (C.3), we can derive the expressions for the crack mouth opening displacement for Euler–Bernoulli beam theory as

D.3 $$\begin{aligned}&\Delta (F) = \frac{2\ F\ {a}_0^3}{3\ EI}, \nonumber \\&\quad \text {before crack propagation } (F\le F_{max}), \end{aligned}$$

D.4 $$\begin{aligned}&\Delta (a) = \frac{2\ F(a)\ a^3}{3\ EI},\nonumber \\&\quad \text {during crack propagation } (a\ge {a}_0), \end{aligned}$$

where

D.5 $$\begin{aligned} F_{max}=\frac{\sqrt{EI\ b\ G_c}}{{a}_0} \quad \text {and} \quad F(a)=\frac{\sqrt{EI\ b\ G_c}}{a}.\nonumber \\ \end{aligned}$$

The last expression gives the widely used LEFM formula for $G_c$ in the case of a DCB with prescribed displacement

D.6 $$\begin{aligned} G_c=\frac{F^2\ a^2}{b\ EI}, \end{aligned}$$

where again, for the sake of simplicity, we replace $F(a)$ simply by $F$. By combining (D.4) and (D.6) we get

D.7 $$\begin{aligned} G_c=\frac{F^2}{b\ EI}\left( \frac{3\ EI\ \Delta }{2\ F}\right) ^{\frac{2}{3}}=\root 3 \of {\frac{9\ \Delta ^2\ F^4}{4\ b^3\ EI}}. \end{aligned}$$

This formula requires the knowledge (experimental measurement) of only $\Delta$ and $F$, whereas the knowledge of the crack length $a$ is not relevant. Note that expression for $G_c$ according to Timoshenko beam theory which requires the knowledge of only $\Delta$ and $F$ can be derived, too, by combining (117) and (115), but this procedure is less-straight forward and it requires solving of a cubic equation. This result is not reported for the sake of simplicity and because in Sect. 7.2 we demonstrate that sufficiently accurate calculation of $G_c$ can be accomplished using formula (D.7).

## Appendix E: Expressions of $v_1$, $v_1^{\prime }$, $\varphi _1$, $\sigma$, ${{\mathcal {T}}}_1$ and ${{\mathcal {M}}}_1$ for the limit case of LEFM

We already mentioned that for the limit case of LEFM ${\delta }_0={\delta }_c\rightarrow 0$ and $\sigma _{max}\rightarrow \infty$, which results in $\lambda \rightarrow \infty$ and $\omega \rightarrow \infty$. This means that only the solution for $v_1(x)$ when $\omega >1$ (see (20)) is considered in the limit case of LEFM, i.e.

E.1 $$\begin{aligned} v_1(x)=e^{-\lambda \zeta _1 x}\ C_1+e^{-\lambda \zeta _2 x}\ C_2. \end{aligned}$$

Constants $C_1$ and $C_2$ are defined according to (31) for a DCB with prescribed rotations, or according to (68) for a DCB with prescribed displacement. In the following subsections we will present the limit (LEFM) values of functions $v_1$, $v_1^{\prime }$, $\varphi _1$, $\sigma$, ${{\mathcal {T}}}_1$ and ${{\mathcal {M}}}_1$ for both cases.

### Appendix E.1: DCB with prescribed rotations

Substituting (31) for $C_1$ and $C_2$ in (E.1) results in

E.2 $$\begin{aligned} v_1(x)= & {} \frac{M}{EI\ \lambda ^2}\left( \frac{e^{-\lambda \zeta _1 x}}{1-\zeta _2^2}+\frac{e^{-\lambda \zeta _2 x}}{1-\zeta _1^2}\right) \nonumber \\= & {} \frac{M}{EI\ \lambda ^2(\zeta _1-\zeta _2)}\left( \zeta _1\ e^{-\lambda \zeta _1 x}-\zeta _2\ e^{-\lambda \zeta _2 x}\right) , \end{aligned}$$

where the relation $\zeta _1 \zeta _2=1$ is used to simplify the expression. If we introduce the following notation

E.3 $$\begin{aligned} \rho _1&= \zeta _1/\lambda =\sqrt{\chi +\sqrt{\chi ^2-1/\lambda ^4}}, \end{aligned}$$

E.4 $$\begin{aligned} \rho _2&= \zeta _2/\lambda =\sqrt{\chi -\sqrt{\chi ^2-1/\lambda ^4}}, \end{aligned}$$

where $\chi =EI/(2 {\mu }{A}_s)$, we can rewrite (E.2) as

E.5 $$\begin{aligned} v_1(x)=\frac{M}{EI\ \lambda ^2(\rho _1-\rho _2)}\left( \rho _1\ e^{-\lambda ^2\rho _1 x}-\rho _2\ e^{-\lambda ^2\rho _2 x} \right) . \end{aligned}$$

Multiplying (E.3) and (E.4) and because $\zeta _1\zeta _2=1$, one then has that $\lambda ^2\rho _2=1/\rho _1$, so that we can write:

E.6 $$\begin{aligned} v_1(x)=\frac{M}{EI\ \lambda ^2(\rho _1-\rho _2)}\left( \rho _1\ e^{-\lambda ^2\rho _1 x}-\rho _2\ e^{-\frac{x}{\rho _1}} \right) . \end{aligned}$$

Note that in the exponent of the second term in the parenthesis we avoid $\lambda ^2\rho _2$ and use $1/\rho _1$ instead because

E.7 $$\begin{aligned} \lim _{\lambda \rightarrow \infty }\rho _1=\sqrt{2\chi }= \sqrt{\frac{EI}{{\mu }{A}_s}} ,\quad \text {and} \quad \lim _{\lambda \rightarrow \infty }\rho _2=0, \end{aligned}$$

show that the limit value of $\lambda ^2\rho _2$, unlike $1/\rho _1$, is less convenient for computer implementation. From (E.6) it then follows that

E.8 $$\begin{aligned} \lim _{\lambda \rightarrow \infty }v_1(x)=0, \quad \text {for} \quad x\ge 0. \end{aligned}$$

According to (89) we can write

E.9 $$\begin{aligned} \sigma (x)=\frac{\sigma _{max}}{{\delta }_0}\delta (x)=\frac{2\ \sigma _{max}}{{\delta }_0}v_1(x), \end{aligned}$$

which after substituting (E.6) and using definition (9)$_1$ for $\lambda$ becomes

E.10 $$\begin{aligned} \sigma (x)=\frac{M\left( \lambda ^2\ \rho _1^2\ e^{-\lambda ^2 \rho _1 x}-e^{-\frac{x}{\rho _1}}\right) }{b\left( \rho _1^2-\frac{1}{\lambda ^2}\right) }. \end{aligned}$$

Therefore, we have $\sigma (0)=M\lambda ^2/b$ and

E.11 $$\begin{aligned} \sigma ^L(0)=\lim _{\lambda \rightarrow \infty }\sigma (0)=\infty , \end{aligned}$$

whereas

E.12 $$\begin{aligned} \sigma ^L(x)= & {} \lim _{\lambda \rightarrow \infty }\sigma (x)\nonumber \\= & {} -\dfrac{M\ {\mu }{A}_s}{b\ EI}e^{-\sqrt{\frac{{\mu }{A}_s}{EI}}x}, \quad \hbox {for }x>0, \end{aligned}$$

from which it follows that

E.13 $$\begin{aligned} \sigma ^L(0^+)=-\dfrac{M\ {\mu }{A}_s}{b\ EI}. \end{aligned}$$

From (E.10) it can be shown that the interface stress profile for finite values of $\lambda$ starts from a positive value at $x=0$, decreases to zero at a coordinate $x=x_0=\ln (\rho _1/\rho _2)/\left[ \lambda ^2(\rho _1-\rho _2)\right]$, continues decreasing to a minimum, negative value, at a coordinate $x_{min}=2x_0$, and then remains negative, but also increases and approaches zero asymptotically for $x\rightarrow \infty$. For $\lambda \rightarrow \infty$, $x_0\rightarrow 0$ and $x_{min}\rightarrow 0$. Stress profiles for different values of $\lambda$ are reported in Fig. 11b. Note that the integral of the stress profile $\sigma ^L(x)$ in (E.12) is finite:

E.14 $$\begin{aligned} \int _{0^+}^\infty \sigma ^L(x) dx=-\frac{M}{b}\sqrt{\frac{{\mu }{A}_s}{EI}} \end{aligned}$$

and therefore, for the vertical equilibrium to be satisfied the resultant $F_0=b\lim \limits _{c\rightarrow 0}\int \limits _0^c \sigma ^L(x) \mathrm {d} x$ of the infinite stress $\sigma ^L(0)$ in (E.11) must be finite:

E.15 $$\begin{aligned} F_0+b\int _{0^+}^\infty \sigma ^L(x) dx=0\quad \Longrightarrow \quad F_0=M\sqrt{\frac{{\mu }{A}_s}{EI}}, \end{aligned}$$

where $F_0$ is a tensile concentrated force at the crack tip pointing downwards. Hence, the LEFM-limit stress profile, $\sigma ^L$, is not a proper function but a generalised one, which is the sum of (E.12) and of $F_0/b$ multiplied by a Dirac distribution ${{\mathcal {D}}}$ centred at zero:

E.16 $$\begin{aligned} \sigma ^L(x)= & {} -\dfrac{M\ {\mu }{A}_s}{b\ EI}e^{-\sqrt{\frac{{\mu }{A}_s}{EI}}x}\nonumber \\&\quad +\frac{M}{b}\sqrt{\frac{{\mu }{A}_s}{EI}}{{\mathcal {D}}}(x), \quad \text {for } x\ge 0. \end{aligned}$$

Thus, according to (5), we can define the distribution of loads on the upper layer in the limit case of LEFM as

E.17 $$\begin{aligned} q^L(x)= & {} -\sigma ^L(x)\ b=\dfrac{M\ {\mu }{A}_s}{EI}e^{-\sqrt{\frac{{\mu }{A}_s}{EI}}x}\nonumber \\&-\,M\sqrt{\frac{{\mu }{A}_s}{EI}} {{\mathcal {D}}}(x), \quad \text {for } x\ge 0. \end{aligned}$$

Because ${{\mathcal {T}}}_1^{\prime }(x)=q(x)$, we can write

E.18 $$\begin{aligned} {{\mathcal {T}}}^L_1(x)= & {} \int q^L(x)\mathrm {d}x+c_1\nonumber \\= & {} -\,M\sqrt{\dfrac{{\mu }{A}_s}{EI}}e^{-\sqrt{\frac{{\mu }{A}_s}{EI}}x}-M\sqrt{\frac{{\mu }{A}_s}{EI}} {{\mathcal {H}}}(x)+c_1, \end{aligned}$$

where

E.19 $$\begin{aligned} {{\mathcal {H}}}(x)=\left\{ \begin{array}{l l} 0 &{} \text {for } x=0,\\ 1 &{} \text {for } x>0, \end{array} \right. \end{aligned}$$

is Heaviside (step) function and $c_1$ is an integration constant. Note that ${{\mathcal {H}}}(x)$ is dimensionless, whereas the dimension of ${{\mathcal {D}}}(x)$ is the inverse of a length. Using the boundary condition ${{\mathcal {T}}}_1^L(0)=0$, we obtain $c_1=F_0=M\sqrt{{\mu }{A}_s/EI}$, so that

E.20 $$\begin{aligned} {{\mathcal {T}}}_1^L(x)= & {} -M\sqrt{\dfrac{{\mu }{A}_s}{EI}}\left[ e^{-\sqrt{\frac{{\mu }{A}_s}{EI}}x}\right. \nonumber \\&\left. +\,({{\mathcal {H}}}(x)-1)\right] , \quad \text {for } x\ge 0, \end{aligned}$$

Because ${{\mathcal {T}}}_1(x)={{\mathcal {M}}}_1^{\prime }(x)$, it follows that the distribution of bending moments on the upper layer for the limit case of LEFM reads

E.21 $$\begin{aligned} {{\mathcal {M}}}_1^L(x)= & {} \int {{\mathcal {T}}}_1^L(x)\mathrm {d}x=Me^{-\sqrt{\frac{{\mu }{A}_s}{EI}}x}\nonumber \\&-M\sqrt{\dfrac{{\mu }{A}_s}{EI}}\int ({{\mathcal {H}}}(x)-1)\mathrm {d}x+c_2, \end{aligned}$$

where $c_2$ is an integration constants. Because $\int ({{\mathcal {H}}}(x)-1)\mathrm {d}x=0$ for any $x\ge 0$, condition ${{\mathcal {M}}}_1^L(0)=M$ gives $c_2=0$, and we finally have

E.22 $$\begin{aligned} {{\mathcal {M}}}_1^L(x)=Me^{-\sqrt{\frac{{\mu }{A}_s}{EI}}x}, \quad \text {for } x\ge 0. \end{aligned}$$

Note that, as expected, ${{\mathcal {T}}}_1^L(\infty )={{\mathcal {M}}}_1^L(\infty )=0$.

From (E.6) we can obtain

E.23 $$\begin{aligned} v_1^{\prime }(x)=-\frac{M\left( \rho _1^2\ e^{-\lambda ^2\rho _1 x}-\rho _2^2\ e^{-\frac{x}{\rho _1}}\right) }{EI(\rho _1-\rho _2)}, \end{aligned}$$

which gives

E.24 $$\begin{aligned} v_1^{\prime }(0)= & {} -\frac{M(\rho _1+\rho _2)}{EI}, \quad \text {and}\nonumber \\ v_1^{\prime L}(0)= & {} \lim \limits _{\lambda \rightarrow \infty }v_1^{\prime }(0)=-\frac{M}{\sqrt{EI\ {\mu }{A}_s}}. \end{aligned}$$

The value of $v_1^{\prime }(x)$ for the limit case of LEFM can be obtained from (E.23) as

E.25 $$\begin{aligned} v_1^{\prime L}(x)=\lim _{\lambda \rightarrow \infty }v_1^{\prime }(x)=0, \quad \text {for } x>0, \end{aligned}$$

which, because $v_1^{\prime L}(0)$ is defined in (E.24), shows that the function $v^{\prime L}(x)$ has a discontinuity at the crack tip ($x=0$), too. Thus, we can write

E.26 $$\begin{aligned} v_1^{\prime L}(x)=\frac{M}{\sqrt{EI\ {\mu }{A}_s}}({{\mathcal {H}}}(x)-1), \quad \text {for } x\ge 0. \end{aligned}$$

Finally, from $\varphi _1(x)=v_1^{\prime }(x)+{{\mathcal {T}}}_1(x)/{\mu }{A}_s$ for the limit case of LEFM we can obtain

E.27 $$\begin{aligned} \varphi _1^L(x)=-\frac{M}{\sqrt{EI\ {\mu }{A}_s}}e^{-\sqrt{\frac{{\mu }{A}_s}{EI}}x}, \quad \text {for } x\ge 0, \end{aligned}$$

which gives the rotation of the upper layer at the crack tip $\varphi _1^L(0)=-M/\sqrt{EI\ {\mu }{A}_s}$.

### Appendix E.2: DCB with prescribed displacement

Substituting (68) in (E.1) gives

E.28 $$\begin{aligned} v_1(x)= & {} \frac{F}{EI\ \lambda ^3(\zeta _1-\zeta _2)}\left[ \zeta _1(\zeta _1+{a}_0\ \lambda )e^{-\lambda \zeta _1 x}\right. \nonumber \\&\left. -\,\zeta _2(\zeta _2+{a}_0\ \lambda )e^{-\lambda \zeta _2 x}\right] , \end{aligned}$$

or

E.29 $$\begin{aligned} v_1(x)= & {} \frac{F}{EI\ \lambda ^2(\rho _1-\rho _2)}\left[ \rho _1(\rho _1+{a}_0)e^{-\lambda ^2\rho _1 x}\right. \nonumber \\&\left. -\,\rho _2(\rho _2+{a}_0)e^{-\frac{x}{\rho _1}}\right] . \end{aligned}$$

Using (E.9) we can obtain

E.30 $$\begin{aligned} \sigma (x)= & {} \frac{F}{b\left( \rho _1-\rho _2\right) }\left( \frac{\rho _1+{a}_0}{\rho _2}\ e^{-\lambda ^2 \rho _1 x}\right. \nonumber \\&\left. -\,\frac{\rho _2+{a}_0}{\rho _1}e^{-\frac{x}{\rho _1}}\right) , \end{aligned}$$

from which it follows that

E.31 $$\begin{aligned} \sigma (0)= & {} \frac{F\ \lambda ^2}{b}\left( {a}_0+\rho _1+\rho _2\right) \quad \text {and} \nonumber \\ \sigma ^L(0)= & {} \lim _{\lambda \rightarrow \infty }\sigma (0)=\infty , \end{aligned}$$

whereas

E.32 $$\begin{aligned} \sigma ^L(x)= & {} \lim _{\lambda \rightarrow \infty }\sigma (x) \nonumber \\= & {} -\dfrac{F\ {a}_0\ {\mu }{A}_s}{b\ EI}e^{-\sqrt{\frac{{\mu }{A}_s}{EI}}x}, \quad \hbox {for }x>0, \end{aligned}$$

so that

E.33 $$\begin{aligned} \sigma ^L(0^+)=-\dfrac{F\ {a}_0\ {\mu }{A}_s}{b\ EI}. \end{aligned}$$

The interface stress profile is now very similar to the one defined in (E.12) for the case of a DCB with prescribed rotations. It can be shown that for (E.32) zero stress and minimum stress correspond to

E.34 $$\begin{aligned}&x_0=\frac{1}{\lambda ^2(\rho _1-\rho _2)}\ln \frac{\rho _1({a}_0+\rho _1)}{\rho _2({a}_0+\rho _2)},\quad \text {and}\nonumber \\&x_{min}=\frac{1}{\lambda ^2(\rho _1-\rho _2)}\ln \frac{\rho _1^2({a}_0+\rho _1)}{\rho _2^2({a}_0+\rho _2)}, \end{aligned}$$

respectively, where as $\lambda \rightarrow \infty$, both $x_0\rightarrow 0$ and $x_{min}\rightarrow 0$. Considering that $F{a}_0$ in (E.33) and $M$ in (E.13) both come from the same moment boundary condition - (30)$_1$ for the case of prescribed rotations and (67)$_1$ for the prescribed displacement - these two results are obviously the same, i.e. $\sigma ^L(0^+)=-{\mu }{A}_s{{\mathcal {M}}}_1(0)/(bEI)$. Again, in the limit case where $\lambda \rightarrow \infty$, the resultant of the tensile stresses at the interface tends to a finite limit value $F_0$. The integral of the limit stress profile $\sigma ^L(x)$ in (E.32) has a finite value which reads

E.35 $$\begin{aligned} \int _{0^+}^\infty \sigma ^L(x) \mathrm {d}x=-\frac{F\ {a}_0}{b}\sqrt{\frac{{\mu }{A}_s}{EI}}, \end{aligned}$$

and therefore, for the vertical equilibrium of the top arm to be satisfied the resultant $F_0=b\lim \limits _{c\rightarrow 0}\int \limits _0^c \sigma ^L(x) \mathrm {d} x$ of $\sigma ^L(0)$ in (E.31) must be finite:

E.36 $$\begin{aligned}&-F+F_0+b\int _0^\infty \sigma ^L(x) \mathrm {d}x=0\quad \nonumber \\&\quad \Longrightarrow F_0=F\left( 1+{a}_0\sqrt{\frac{{\mu }{A}_s}{EI}}\right) , \end{aligned}$$

where $F_0$ is a tensile concentrated force at the crack tip pointing downwards. Note that using (30) and (67), (E.15) and (E.36) may be uniquely expressed as $F_0={{\mathcal {T}}}_1(0)+{{\mathcal {M}}}_1(0)\sqrt{{\mu }{A}_s/EI}$. Thus, similar as in (E.16), we can write

E.37 $$\begin{aligned} \sigma ^L(x)= & {} -\dfrac{F\ {a}_0\ {\mu }{A}_s}{b\ EI}e^{-\sqrt{\frac{{\mu }{A}_s}{EI}}x}\nonumber \\&+\frac{F}{b}\left( 1+{a}_0\sqrt{\frac{{\mu }{A}_s}{EI}}\right) {{\mathcal {D}}}(x), \quad \text {for } x\ge 0, \end{aligned}$$

and from there $q^L(x)=-\sigma ^L(x)\ b$ gives

E.38 $$\begin{aligned} q^L(x)= & {} \dfrac{F\ {a}_0\ {\mu }{A}_s}{EI}e^{-\sqrt{\frac{{\mu }{A}_s}{EI}}x}\nonumber \\&-F\left( 1+{a}_0\sqrt{\frac{{\mu }{A}_s}{EI}}\right) \ {{\mathcal {D}}}(x), \quad \text {for } x\ge 0. \end{aligned}$$

We can then write

E.39 $$\begin{aligned} {{\mathcal {T}}}^L_1(x)= & {} \int q^L(x)\mathrm {d}x+c_1=-F\ {a}_0\sqrt{\dfrac{{\mu }{A}_s}{EI}}e^{-\sqrt{\frac{{\mu }{A}_s}{EI}}x}\nonumber \\&-\,F\left( 1+{a}_0\sqrt{\frac{{\mu }{A}_s}{EI}}\right) \ {{\mathcal {H}}}(x)+c_1. \end{aligned}$$

From ${{\mathcal {T}}}_1^L(0)=0$ we obtain $c_1=F_0=F(1+{a}_0\sqrt{{\mu }{A}_s/EI})$ so that

E.40 $$\begin{aligned} {{\mathcal {T}}}_1^L(x)= & {} -F\ {a}_0\sqrt{\dfrac{{\mu }{A}_s}{EI}}\left[ e^{-\sqrt{\frac{{\mu }{A}_s}{EI}}x}+({{\mathcal {H}}}(x)-1)\right] \nonumber \\&-\,F({{\mathcal {H}}}(x)-1), \quad \text {for } x\ge 0. \end{aligned}$$

From here, by taking into account that $\int ({{\mathcal {H}}}(x)-1)\mathrm {d}x=0$ for any $x\ge 0$, we obtain

E.41 $$\begin{aligned} {{\mathcal {M}}}_1^L(x)=\int {{\mathcal {T}}}_1^L(x)\mathrm {d}x=F\ {a}_0\ e^{-\sqrt{\frac{{\mu }{A}_s}{EI}}x}, \end{aligned}$$

where the integration constant $c_2$ vanishes due to ${{\mathcal {M}}}_1^L(0)=F{a}_0$. Note that, as expected, ${{\mathcal {T}}}_1^L(\infty )={{\mathcal {M}}}_1^L(\infty )=0$.

From (E.29) we can obtain

E.42 $$\begin{aligned}&v_1^{\prime }(x)\nonumber \\&\quad =-\frac{F\left[ \rho _1^2(\rho _1+{a}_0) e^{-\lambda ^2\rho _1 x}-\rho _2^2(\rho _2+{a}_0) e^{-\frac{x}{\rho _1}}\right] }{EI(\rho _1-\rho _2)}, \end{aligned}$$

which gives

E.43 $$\begin{aligned} v_1^{\prime }(0)=-\frac{F}{EI}\left[ \frac{EI}{{\mu }{A}_s}+\frac{1}{\lambda ^2}+{a}_0(\rho _1+\rho _2)\right] , \end{aligned}$$

and

E.44 $$\begin{aligned} v_1^{\prime L}(0)=\lim \limits _{\lambda \rightarrow \infty }v_1^{\prime }(0)=-\frac{F}{{\mu }{A}_s}-\frac{F\ {a}_0}{\sqrt{EI\ {\mu }{A}_s}}. \end{aligned}$$

The value of $v_1^{\prime }(x)$ for the limit case of LEFM is obtained from (E.42) as

E.45 $$\begin{aligned} v_1^{\prime L}(x)=\lim _{\lambda \rightarrow \infty }v_1^{\prime }(x)=0, \quad \text {for } x>0, \end{aligned}$$

which shows that the function $v_1^{\prime L}(x)$ has a discontinuity at the crack tip ($x=0$). Thus, we can write

E.46 $$\begin{aligned} v_1^{\prime L}(x)= & {} \left( \frac{F}{{\mu }{A}_s}+\frac{F\ {a}_0}{\sqrt{EI\ {\mu }{A}_s}}\right) \nonumber \\&\quad \times ({{\mathcal {H}}}(x)-1), \quad \text {for } x\ge 0. \end{aligned}$$

We can finally obtain

E.47 $$\begin{aligned} \varphi _1^L(x)= & {} v_1^{\prime L}+\frac{{{\mathcal {T}}}_1^L(x)}{{\mu }{A}_s}\nonumber \\= & {} -\frac{F\ {a}_0}{\sqrt{EI\ {\mu }{A}_s}}e^{-\sqrt{\frac{{\mu }{A}_s}{EI}}x}, \quad \text {for } x\ge 0, \end{aligned}$$

where the rotation of the upper layer at the crack tip is $\varphi _1^L(0)=-F{a}_0/\sqrt{EI{\mu }{A}_s}$.

### Appendix E.3: Euler–Bernoulli beam theory

In the limit case of LEFM for Euler–Bernoulli beam theory ($\mu \rightarrow \infty$) Eqs. (98)–(103) for a DCB with prescribed rotations become

E.48 $$\begin{aligned} v_1^L(x)&= v_1^{\prime L}(x)= \varphi _1^L(x)= {{\mathcal {T}}}_1^L(x)=0,\end{aligned}$$

E.49 $$\begin{aligned} \sigma ^L(x)&= {{\mathcal {D}}}(x),\end{aligned}$$

E.50 $$\begin{aligned} {{\mathcal {M}}}_1^L(x)&= M(1-{{\mathcal {H}}}(x)). \end{aligned}$$

Equations (107)–(112) for a DCB with prescribed displacement become

E.51 $$\begin{aligned} v_1^L(x)&= v_1^{\prime L}(x)= \varphi _1^L(x)=0, \end{aligned}$$

E.52 $$\begin{aligned} \sigma ^L(x)&= {{\mathcal {D}}}(x), \end{aligned}$$

E.53 $$\begin{aligned} {{\mathcal {T}}}_1^L(x)&= F\ (1-{{\mathcal {H}}}(x)), \end{aligned}$$

E.54 $$\begin{aligned} {{\mathcal {M}}}_1^L(x)&= F\ {a}_0(1-{{\mathcal {H}}}(x)). \end{aligned}$$

These expressions confirm that the DCB arms do not deform at and in front of the crack tip when they are modelled as Euler–Bernoulli beams. Analogous expressions for the phase of crack propagation could be obtained by substituting $M$ with ${M}_{max}$, $F$ with $F(a)$ and ${a}_0$ with $a$.

## List of symbols

*A*: Cross-sectional area of a single DCB arm
*a*: Crack length
$a_0$: Initial crack length
*b*: Width of a DCB
$C_i$: Integration constants for the undamaged part ($i=1,\ldots ,6$)
$C^j_i$: Constants depending on $C_3$, $C_4$, $\xi _3$ and $\xi _4$ ($i=3,4$ and $j=M,T$)
${\overline{C}}_i$: Integration constants for the undamaged part that are zero because of the boundary conditions for a semi-infinite DCB ($i=1,\ldots ,6$)
$$c_2$$: function $$\cos (L_{cz}\kappa \xi _2)$$
$${\overline{c}_2}$$: function $$\cos (\overline{L}_{cz} \kappa \xi _2)$$
$$ch_1$$: function $$\cosh (L_{cz}\kappa \xi _1)$$
$${\overline{ch}_1}$$: function $$\cosh (\overline{L}_{cz}\kappa \xi _1)$$
$${{\mathcal {D}}}$$: Dirac delta
$D_0$: A constant depending on $\psi$
$D_i$: Integration constants for the damaged part ($i=1,\ldots ,4$)
$$D_{ij} $$: Constants for the damaged part depending on the value of $$\omega $$ ($$i=1,\ldots ,4$$ and $$j=1,2$$)
$E$: Young’s modulus of DCB arms
$F$: Vertical force applied on the upper layer of a DCB with prescribed displacement
$F_0$: A tensile stress resultant (concentrated force) at the crack tip for the LEFM limit case
$F_E$: Value of the applied force computed using Euler–Bernoulli beam theory
$F_L$: Maximum value of the applied force in the linear-elastic phase for a DCB with prescribed displacement
$F_{max}$: Maximum value of the applied load for a DCB with prescribed displacement
$F_T$: Value of the applied force computed using Timoshenko beam theory
$F_{\sigma _{max}}$: Value of the applied force computed for a finite value of $\sigma _{max}$
$F_{\infty }$: Value of the applied force for the LEFM limit
$G_c$: Critical energy release rate
$${{\mathcal {H}}}$$: Heaviside function
$h$: Depth of a single DCB arm
*I*: Second moment of area of the cross-section of a single DCB arm
$J_c$: Critical value of the J integral
$k_s$: Shear correction coefficient
*L*: Total length of a DCB specimen
$L_{cz}$: Length of the cohesive or damage-process zone
${\overline{L}}_{cz}$: Maximum value of $L_{cz}$ for a DCB with prescribed rotations
$L_{cz}^{max}$: Maximum value of $L_{cz}$ for a DCB with prescribed displacement
$L_{cz}^{min}$: Minimum value of $L_{cz}$ during crack propagation for a DCB with prescribed displacement
$M$: Concentrated moment applied on a DCB arm
${{\mathcal {M}}}_1$: Bending moment in the upper DCB arm on the part where the interface is undamaged
${{\mathcal {M}}}_1^L$: LEFM limit value of ${{\mathcal {M}}}_1$
${{\mathcal {M}}}_2$: Bending moment in the upper DCB arm on the part where the interface is damaged
$M_L$: Maximum value of the applied moment in the linear-elastic phase
${M}_{max}$: Maximum value of the applied moment
$q$: Distributed transverse loading along the upper DCB arm
$r_i$: Roots of the characteristic equation for the undamaged part ($i=1,\ldots ,4$)
$$s_2$$: function $$\sin (L_{cz}\kappa \xi _2)$$
$${\overline{s}_2}$$: function $$\sin (\overline{L}_{cz}\kappa \xi _2)$$
$$sh_1$$: function $$\sinh (L_{cz}\kappa \xi _1)$$
$${\overline{sh}_1}$$: function $$\sinh (\overline{L}_{cz} \kappa \xi _1)$$
${{\mathcal {T}}}$: Shear force in the upper DCB arm
${{\mathcal {T}}}_1$: Shear force in the upper DCB arm on the part where the interface is undamaged
${{\mathcal {T}}}_1^L$: LEFM limit value of ${{\mathcal {T}}}_1$
${{\mathcal {T}}}_2$: Shear force in the upper DCB arm on the part where the interface is damaged
$t_i$: Roots of the characteristic equation for the damaged part ($i=1,\ldots ,4$)
$v$: Transversal displacement of the upper DCB arm
$v_1$: Transversal displacement of the upper DCB arm on the part where the interface is undamaged
$v_1^L$: LEFM limit value of $v_1$
$v_2$: Transversal displacement of the upper DCB arm on the part where the interface is damaged
*x*: Co-ordinate along the interface
$x_0$: Co-ordinate *x* corresponding to zero stress at the interface for the case of a linear-elastic interface with brittle failure
$x_{min}$: Co-ordinate *x* corresponding to the minimum (maximum compressive) stress at the interface for the case of a linear-elastic interface with brittle failure
$\alpha$: A constant defined as $\alpha ={\delta }_0/{\delta }_c$
$\beta _i$: Constants depending on $\xi _j$, $D_{kl}$, $\kappa$ and ${a}_0$ ($i=1,\ldots ,8$, $j=1,2$, $k=1,\ldots ,4$ and $l=1,2$)
$\gamma$: Shear strain in the upper DCB arm
$\Delta$: Prescribed crack mouth opening displacement of a DCB
$\Delta _{a}$: Part of the crack mouth opening displacement due to bending of the arms assuming that they are clamped at the crack tip
$\Delta _{CT}^{\delta }$: Part of the crack mouth opening displacement due to the opening at the crack tip
$\Delta _{CT}^{\varphi }$: Part of the crack mouth opening displacement due to the rotation at the crack tip
$\delta$: Relative displacement at the interface in mode I
${\delta }_0$: Linear-elastic limit value of the relative displacement at the interface in mode I
${\delta }_c$: Relative displacement at the interface corresponding to the total loss of interconnection in mode I
$\varepsilon _r$: Relative error due to using Euler–Bernoulli instead of Timoshenko beam theory
${\overline{\varepsilon }_r}$: Relative difference between CCM and LEFM solutions
$\zeta _i$: Constants depending on $\omega$ ($i=1,\ldots ,4$)
${\overline{\zeta }_i}$: Constants depending on $\omega$ ($i=1,\ldots ,4$)
$\eta$: A constant defined as $\eta =\lambda /\kappa$
$\eta _E$: A constant defined as $\eta _E=\lambda _E/\kappa$
$\theta$: Prescribed rotation on a DCB arm
$\kappa$: A constant depending on the bending stiffness of DCB arms and the softening part of the $\sigma -\delta$ traction-separation law of the interface
$\lambda$: A constant depending on the bending stiffness of DCB arms and the linear-elastic stiffness of the interface
$\lambda _E$: A constant defined as $\lambda _E=\sqrt{2}\lambda /2$
$\mu$: Shear modulus of DCB arms
$\nu$: Poisson’s ratio of DCB arms
$\xi _i$: Constants depending on $\psi$ ($i=1,2$)
$\rho _i$: Constants defined as $\rho _i=\zeta _i/\lambda$ ($i=1,2$)
$\sigma$: Contact traction at the interface in mode I
$\sigma ^L$: LEFM limit value of $\sigma$
$\sigma _{max}$: Maximum value of contact tractions at the interface in mode I
$\varphi$: Cross-sectional rotation of the upper DCB arm
$\varphi _1$: Cross-sectional rotation of the upper DCB arm on the part where the interface is undamaged
$\varphi _2$: Cross-sectional rotation of the upper DCB arm on the part where the interface is damaged
$\varphi _1^L$: LEFM limit value of $\varphi _1$
$\chi$: A constant defined as $\chi =EI/(2{\mu }{A}_s)$
$\psi$: A constant depending on bending and shear stiffness of DCB arms, and $\kappa$
$\omega$: A constant depending on bending and shear stiffness of DCB arms, and $\lambda$

## List of Abbreviations

ASTM: American Society for Testing and Materials
BS: British Standard
CBBM: Compliance-based beam method
CBT: Corrected beam theory
CCM: Cohesive crack model
CZM: Cohesive zone model
DCB: Double cantilever beam
EBT: Enhanced beam theory
ESBT: Enhanced simple beam theory
FE: Finite element
FEA: Finite element analysis
ISO: International Organization for Standardization
LEFM: Linear elastic fracture mechanics
SBT: Simple beam theory
TDCB: Tapered double cantilever beam
